# Immunoproteasome subunit ß5i/LMP7-deficiency in atherosclerosis

**DOI:** 10.1038/s41598-017-13592-w

**Published:** 2017-10-17

**Authors:** Bernd Hewing, Antje Ludwig, Cristian Dan, Max Pötzsch, Carmen Hannemann, Andreas Petry, Dilyara Lauer, Agnes Görlach, Elena Kaschina, Dominik N. Müller, Gert Baumann, Verena Stangl, Karl Stangl, Nicola Wilck

**Affiliations:** 1 Medizinische Klinik m.S. Kardiologie und Angiologie, Charité-Universitätsmedizin Berlin, Campus Mitte, Berlin, Germany; 2 DZHK (German Centre for Cardiovascular Research), partner site Berlin, Berlin, Germany; 3 Experimental and Molecular Pediatric Cardiology, German Heart Center Munich, Technical University Munich, Munich, Germany; 4 DZHK (German Centre for Cardiovascular Research), partner site Munich, Munich, Germany; 5 Institute of Pharmacology, Center for Cardiovascular Research, Charité-Universitätsmedizin Berlin, Berlin, Germany; 6 Experimental and Clinical Research Center, a joint cooperation of Max Delbrück Center for Molecular Medicine and Charité Medical Faculty, Berlin, Germany; 7 Berlin Institute of Health (BIH), Berlin, Germany; 8 Max Delbrück Center for Molecular Medicine in the Helmholtz Association, Berlin, Germany

## Abstract

Management of protein homeostasis by the ubiquitin-proteasome system is critical for atherosclerosis development. Recent studies showed controversial results on the role of immunoproteasome (IP) subunit β5i/LMP7 in maintenance of protein homeostasis under cytokine induced oxidative stress. The present study aimed to investigate the effect of β5i/LMP7-deficiency on the initiation and progression of atherosclerosis as a chronic inflammatory, immune cell driven disease. LDLR^−/−^LMP7^−/−^ and LDLR^−/−^ mice were fed a Western-type diet for either 6 or 24 weeks to induce early and advanced stage atherosclerosis, respectively. Lesion burden was similar between genotypes in both stages. Macrophage content and abundance of polyubiquitin conjugates in aortic root plaques were unaltered by β5i/LMP7-deficiency. *In vitro* experiments using bone marrow-derived macrophages (BMDM) showed that β5i/LMP7-deficiency did not influence macrophage polarization or accumulation of polyubiquitinated proteins and cell survival upon hydrogen peroxide and interferon-γ treatment. Analyses of proteasome core particle composition by Western blot revealed incorporation of standard proteasome subunits in β5i/LMP7-deficient BMDM and spleen. Chymotrypsin-, trypsin- and caspase-like activities assessed by using short fluorogenic peptides in BMDM whole cell lysates were similar in both genotypes. Taken together, deficiency of IP subunit β5i/LMP7 does not disturb protein homeostasis and does not aggravate atherogenesis in LDLR^−/−^ mice.

## Introduction

Dysregulation of the ubiquitin-proteasome system (UPS) has been associated with atherosclerosis development^1–3^. The UPS is essential for the intracellular degradation of proteins in all eukaryotic cells. Proteolytic degradation takes place in the 26 S core proteasome, a multicatalytic peptidase carrying standard chymotrypsin-like (β5 subunit), trypsin-like (β2 subunit) and caspase-like (β1 subunit) activities. Under cytokine stress these standard catalytic activities of the proteasome are replaced by distinct isoforms (β5i/LMP7, β1i/LMP2 and  β2i/MECL-1), leading to the formation of the immunoproteasome (IP)^4^. Moreover, replacement of solely one or two standard β subunits results in the formation of intermediate-type proteasomes, which have been detected in various tissues^5^. Whereas non-immune cells predominantly express standard proteasomes, IPs are the predominant proteasomes in leukocytes. The IP is long known to mediate immune responses, as it efficiently generates peptides for MHC class I restricted antigen presentation^6^.

An increasing number of reports indicate an important role of IP subunit β5i/LMP7 in inflammatory diseases. Hereditary inflammatory disorders have been linked to mutations in the β5i/LMP7 gene^7–10^. Selective pharmacological inhibition of β5i/LMP7 showed anti-inflammatory effects in several experimental inflammatory models^11–13^. Besides its function in immune responses, a recent study highlighted an involvement of β5i/LMP7 in the management of protein homeostasis under cytokine-induced oxidative stress^14^. It was shown that β5i/LMP7-deficiency leads to the formation of intracellular protein aggregates and aggravates experimental autoimmune encephalomyelitis. Interestingly, these finding were challenged by others using similar methods^15^. Yet, given its proposed significance for inflammation and cellular protein homeostasis, we questioned the impact of β5i/LMP7 on atherosclerosis development. Atherosclerosis represents an inflammatory, immune cell driven disease^16^ and defective removal of protein aggregates was proposed to aggravate atherosclerosis development^17^. However, studies investigating the impact of β5i/LMP7-deficiency on atherosclerosis are currently not available.

Therefore, the present study aims at elucidating the effect of β5i/LMP7-deficiency on the composition and function of the UPS in macrophages and its consequences for atherosclerosis as a chronic inflammatory, immune cell driven disease.

## Results

### β5i/LMP7-deficient atherosclerosis mouse model

In order to study the effect of β5i/LMP7-deficiency on atherosclerosis, we generated low-density lipoprotein receptor-deficient and β5i/LMP7-deficient mice (LDLR^−/−^LMP7^−/−^); LDLR^−/−^ littermate mice served as controls. Upon control diet for 6 or 24 weeks, respectively, serum levels of total cholesterol (TC), high-density lipoprotein cholesterol (HDL-C) and non-HDL-C were similar between both genotypes (Supplement Table S1). Serum levels of triglycerides were lower in LDLR^−/−^LMP7^−/−^ mice compared to LDLR^−/−^ littermates solely at 6 weeks of control diet and were found to be equal at 24 weeks of control diet (Supplement Table S1). Serum glucose levels were similar between both genotypes (Fig. S1). Body weight was similar after 6 weeks of diet, but lower in the group of LDLR^−/−^LMP7^−/−^ mice after 24 weeks compared to LDLR^−/−^ littermates (Supplement Table S1). β5i/LMP7-deficiency did not influence cardiac dimensions and function as assessed by echocardiography (Supplement Table S2).

Feeding of a high-fat Western-type diet (WD) for 6 or 24 weeks, respectively, resulted in hyperlipidemia with similar serum levels of TC, HDL-C, non-HDL-C, and glucose between both genotypes (Table 1, Fig. S1). LDLR^−/−^LMP7^−/−^ mice exhibited a trend to higher levels of triglycerides compared to LDLR^−/−^ littermates, which was statistically significant solely after 6 weeks of WD (Table 1). Body weight was lower in LDLR^−/−^ mice (statistically significant after 6 weeks of WD), while the actual body weight gain over the course of the WD did not differ between both genotypes (Table 1). Serum levels of lipid peroxidation products measured as thiobarbituric acid reactive substances (TBARS) were similar in both genotypes (Fig. S2).

**Table 1:** Body weight and lipid levels.

	6 weeks WD		*p*	24 weeks WD		*p*
	LDLR^−/−^	LDLR^−/−^LMP7^−/−^		LDLR^−/−^	LDLR^−/−^LMP7^−/−^	
Body weight, g	31.2 ± 1.5	29.4 ± 2.2	0.045	36.6 ± 4.5	34.6 ± 5.5	0.36
Body weight gain, g	6.6 ± 0.2	6.4 ± 0.8	0.732	12.1 ± 2.4	10.0 ± 3.9	0.323
TC, mg/dl	1318.7 ± 138.6	1281.2 ± 181.0	0.591	1289.5 ± 265.4	1398.4 ± 417.1	0.47
HDL-C, mg/dl	127.7 ± 23.4	135.6 ± 24.7	0.448	247.6 ± 74.6	247.1 ± 78.3	0.989
Non-HDL-C, mg/dl	1191.0 ± 135.6	1145.6 ± 168.3	0.494	1041.9 ± 232.8	1151.3 ± 376.4	0.417
Triglycerides, mg/dl	539.7 ± 165.7	708.2 ± 187.3	0.023	393.9 ± 155.6	482.4 ± 195.8	0.26

n = 11 per group, except LDLR^−/−^LMP7^−/−^ 24 weeks WD n = 12; data are shown as mean ± SD. Statistical analysis using unpaired *t*-test or Mann-Whitney *U* test. WD, Western-type diet; TC, total cholesterol; HDL-C, high-density lipoprotein cholesterol.

### β5i/LMP7-deficiency does not aggravate atherosclerotic lesion progression in LDLR^−/−^ mice

**Figure 1:**
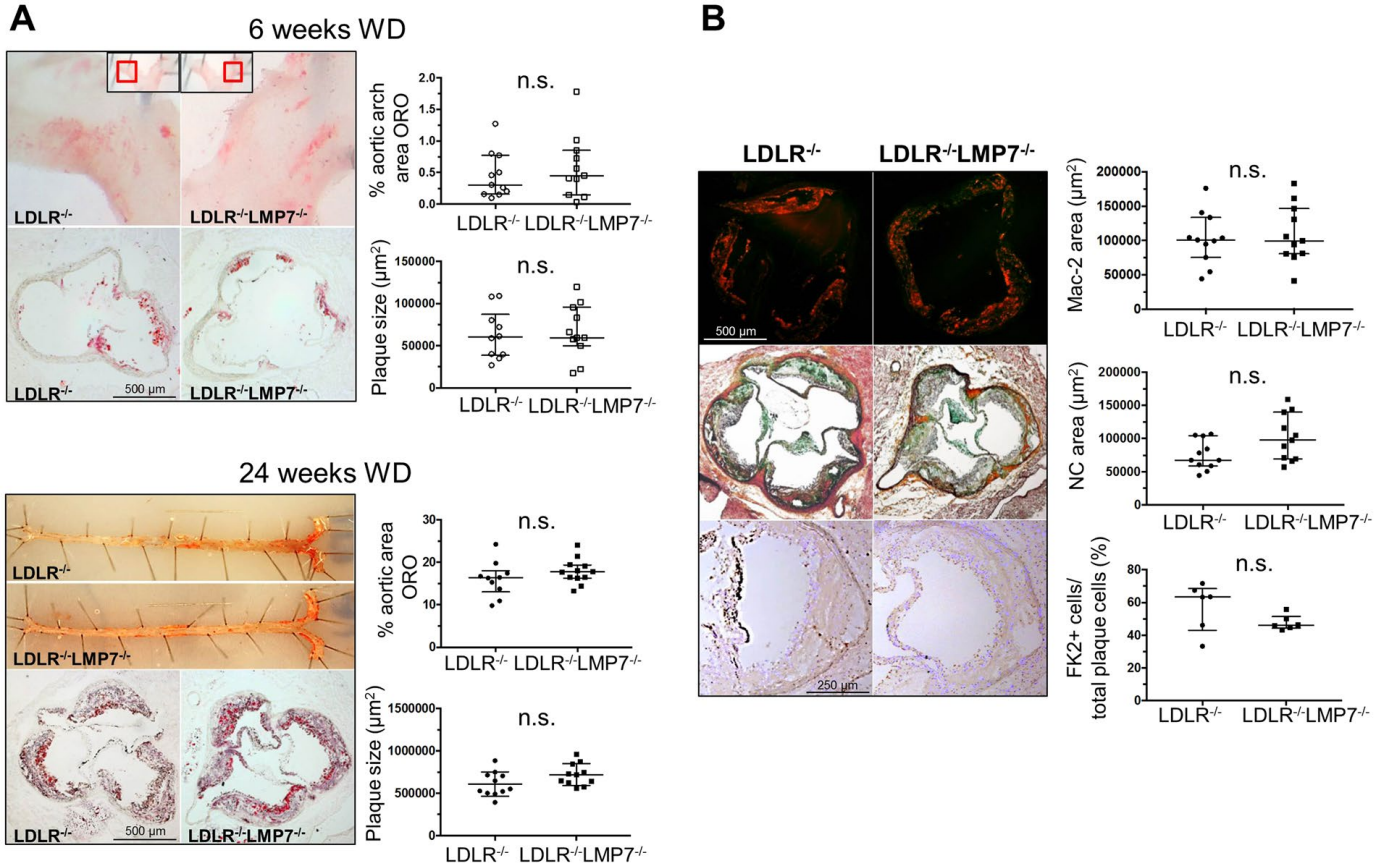
β5i/LMP7-deficiency does not aggravate atherosclerotic lesion progression in LDLR^−/−^ mice. LDLR^−/−^LMP7^−/−^ and LDLR^−/−^ littermate mice were fed a high-fat Western-type diet (WD) for either 6 or 24 weeks to induce early and advanced stage atherosclerosis, respectively. (**A**) Representative *en face* stainings of aortic arch/whole aorta (upper rows) and Oil Red O stainings with hematoxylin counterstain of aortic root cross-sections (bottom rows) for early (upper panels) and advanced (lower panels) stage atherosclerosis with quantification of lesion sizes. (**B)** Representative stainings and quantifications of (upper row) macrophages (MAC-2), (middle row) necrotic core (NC; Movat pentachrome staining), and (bottom row) ubiquitinated proteins (FK2; magnification: 10x) of aortic root cross-sections of advanced stage atherosclerosis. Data in graphs are presented as individual values with median and interquartile ranges indicated. n.s. = statistically non-significant. Unpaired *t*-test (Welch test for unequal variances) or Mann-Whitney *U* test.

LDLR^−/−^LMP7^−/−^ and LDLR^−/−^ littermate mice developed early lesions in atherosclerosis-prone sites such as the aortic root and arch after being fed a WD for 6 weeks. Mice fed a WD over 24 weeks exhibited advanced lesions in the entire aorta, as shown by *en face* and histologic analyses of the aortic root and the *truncus brachiocephalicus* (Figs 1A and S3). Lesion quantification did not reveal significant differences in atherosclerotic plaque burden between both genotypes at either stage of atherosclerosis (Fig. 1A). Plaque macrophage (Mac-2) and T cell (CD4) content in advanced aortic root plaques did not differ between both genotypes (Figs 1B and S4). Cells associated with strong FK2 staining, a marker for accumulation of ubiquitinated proteins, were detectable in macrophage-rich areas and were not significantly affected by β5i/LMP7-deficiency (Fig. 1B). Necrotic core size was similar between both genotypes (Fig. 1B).

### β5i/LMP7-deficiency does not influence macrophage polarization and stress response in BMDM of LDLR^−/−^ mice

**Figure 2:**
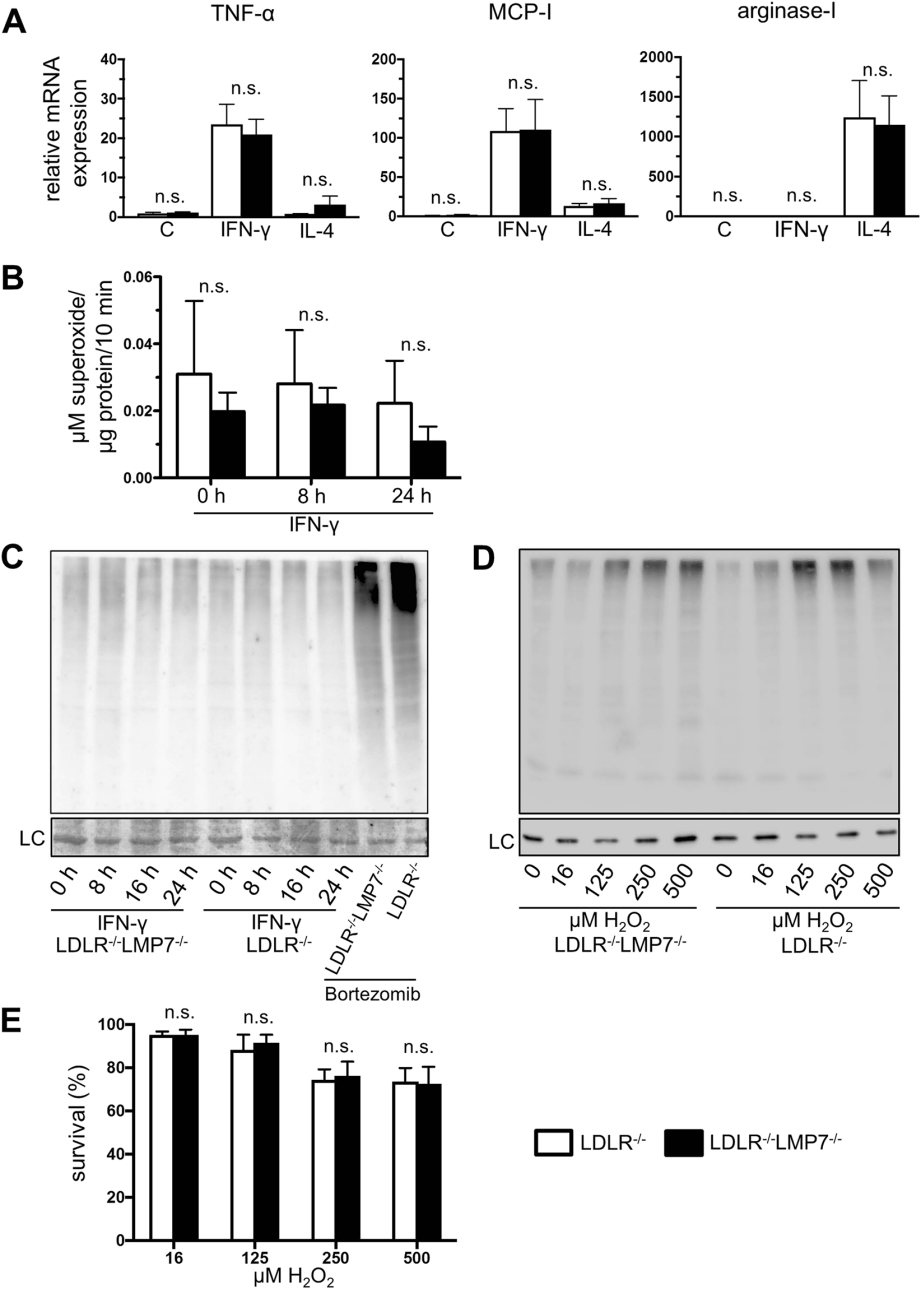
β5i/LMP7-deficiency does not influence macrophage polarization and stress response in BMDM of LDLR^−/−^ mice. BMDM isolated from LDLR^−/−^LMP7^−/−^ and LDLR^−/−^ littermate mice: **(A)** M1/M2 polarization – mRNA expression of tumor necrosis factor-α (TNF-α), monocyte chemoattractant protein-1 (MCP-1) and arginase-1 determined by quantitative real-time RT-PCR after treatment of BMDM with IFN-γ (100 U/ml) and IL-4 (4 U/ml) over 6 hours for M1 and M2 macrophage phenotype polarization, respectively; C = untreated cells; graphs show fold-change in mRNA expression levels relative to untreated BMDM of LDLR^−/−^ mice; n = 5 experiments. Unpaired *t*-test (Welch test for unequal variances) or Mann-Whitney *U* test. (**B)** Superoxide production of IFN-γ-treated (100 U/ml) BMDM determined by electron paramagnetic resonance (EPR) technique; n = 5–7 per group. Two-way ANOVA followed by Sidak’s multiple comparisons test. (**C)** Representative Western blot with anti-ubiquitin antibody of lysates of IFN-γ (100 U/ml) treated BMDM; Bortezomib (5 ng/ml) treated BMDM served as positive controls; n = 4 experiments; LC indicates amidoblack staining as loading control. (**D)** Representative Western blot with anti-ubiquitin antibody of lysates of BMDM after treatment with hydrogen peroxide (H_2_O_2_) over 1 hour; n = 3 experiments. LC indicates GAPDH staining as loading control. **(E**) Survival of BMDM treated with H_2_O_2_ over 1 hour; n = 4 experiments. Kruskal-Wallis test with post hoc comparison by Dunn’s multiple comparison test. Data in graphs are presented as mean ± SEM. n.s. = statistically non-significant.

Macrophage activation states have been shown to differentially influence atherosclerosis^18^. Therefore, we assessed the influence of β5i/LMP7-deficiency on BMDM polarization towards M1 and M2 phenotypes induced by IFN-γ and IL-4, respectively. Figure 2A shows that IFN-γ induced mRNA levels of the M1 marker genes tumor necrosis factor-α (TNF-α) and monocyte chemoattractant protein-1 (MCP-1) to a similar extent in BMDM from LDLR^−/−^LMP7^−/−^ and LDLR^−/−^ littermate mice. Likewise, superoxide anion production of IFN-γ stimulated BMDM was not affected by β5i/LMP7-deficiency (Fig. 2B). IL-4 stimulation induced mRNA expression of M2 marker arginase-1 similarly in BMDM of both genotypes (Fig. 2A).

Next, we determined the influence of cytokine-stimulation and oxidative stress on the content of polyubiquitin conjugates in BMDM by Western blot. The amount of polyubiquitinated proteins was not changed after IFN-γ stimulation up to 24 hours in BMDM of both genotypes (Figs 2C and S5A). After hydrogen peroxide (H_2_O_2_) challenge, we detected a similar increase in polyubiquitinated proteins in BMDM of both genotypes (Figs 2D and S5B). BMDM survival following H_2_O_2_ challenge was similar for both genotypes (Fig. 2E).

### Impact of β5i/LMP7-deficiency on proteasome composition and proteolytic activity in cells and tissues of LDLR^−/−^ mice

**Figure 3:**
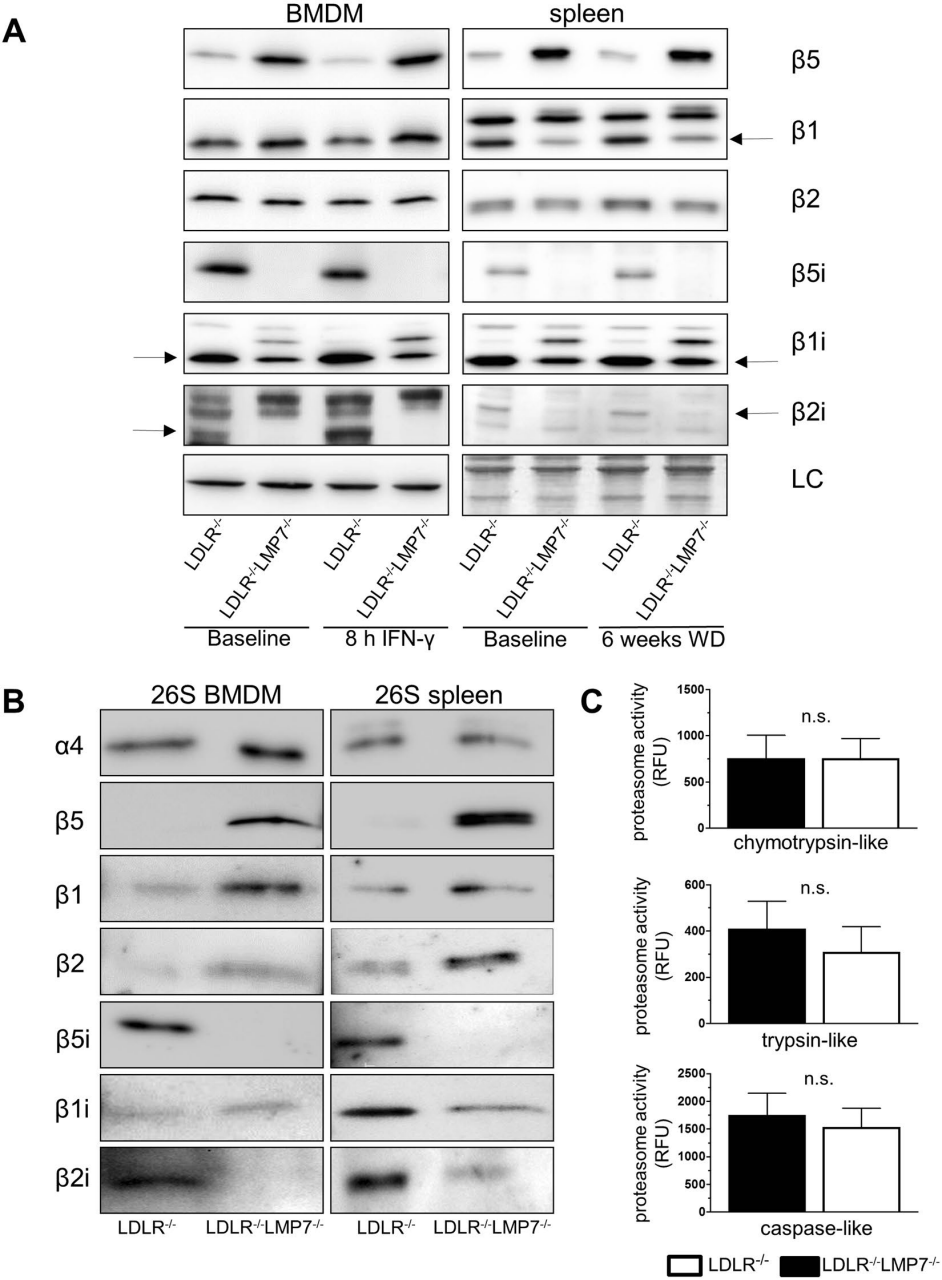
Impact of β5i/LMP7-deficiency on proteasome composition and proteolytic activity in cells and tissues of LDLR^−/−^ mice. **(A**) Western blot analysis of standard proteasome (β1, β2, and β5) and immunoproteasome (β1i, β2i, and β5i) subunit expression in BMDM of LDLR^−/−^LMP7^−/−^ and LDLR^−/−^ at baseline and after treatment with IFN-γ (100 U/ml) over 8 hours (left panel). Right panel shows Western blot analyses of pooled spleen protein samples from LDLR^−/−^LMP7^−/−^ and LDLR^−/−^ mice at baseline and after feeding a high-fat Western-type diet (WD) over 6 weeks (n = 11 mice per group). LC indicates actin staining (for BMDM) or amidoblack staining (for spleen) as loading controls. **(B**) Representative Western blots of standard proteasome and immunoproteasome subunits of isolated 26 S proteasome derived from murine LDLR^−/−^LMP7^−/−^ and LDLR^−/−^ BMDM (left panel) and spleen (right panel) lysates. **(C)** Chymotrypsin-, trypsin- and caspase-like proteasome activities (expressed as relative fluorescence units, RFU) of murine LDLR^−/−^LMP7^−/−^ and LDLR^−/−^ BMDM; n = 4 experiments. Unpaired *t*-test or Mann-Whitney *U* test. Data in graphs are presented as mean ± SEM.

To determine the impact of β5i/LMP7-deficiency on proteasome subunit composition, we performed Western blots in BMDM, spleen and liver lysates of LDLR^−/−^LMP7^−/−^ and LDLR^−/−^ littermate mice. In BMDM lysates from LDLR^−/−^ mice we detected expression of all standard and IP β subunits (Fig. 3A). Standard proteasome subunits β5, β2, β1 and mature forms of IP subunit β1i were detectable in β5i/LMP7-deficient BMDM (under native and IFN-γ stimulated conditions) and in splenic lysates of β5i/LMP7-deficient mice (Fig. 3A). Mature forms of IP subunit β2i were detectable in splenic lysates of β5i/LMP7-deficient mice. In β5i/LMP7-deficient fibroblasts standard proteasome subunits β5, β2, β1 and induction of IP subunit β1i after IFN-γ stimulation were detectable (Fig. S6). Enhanced bands for β5i/LMP7 were observable in spleens and livers of LDLR^−/−^ mice fed a WD compared to tissues from CD fed mice (baseline) indicative of a systemic inflammatory response under WD (Figs 3A and S6).

To examine the composition of β subunits incorporated into proteasomal core particles, we isolated 26 S proteasomes from BMDM and splenic lysates using native gel electrophoresis and determined their subunit composition by Western blot (Fig. 3B). BMDM and spleens of LDLR^−/−^ mice assembled IP subunits β5i/LMP7, β2i, β1i and standard proteasome subunits β1 and β2, indicating the presence of immuno- and intermediate proteasomes. In proteasomes of β5i/LMP7-deficient BMDM and spleen standard subunits β5, β2, β1 and IP subunit β1i (and β2i in spleen) were detectable indicating the presence of standard and intermediate proteasomes. Incorporated β2i was hardly detectable in β5i/LMP7-deficient BMDM. Except for β5i/LMP7, mRNA expression levels of proteasome subunits in BMDM did not differ between both genotypes (Fig. S7).

Next, we determined whether alterations in proteasomal composition ultimately lead to alterations in proteasomal proteolytic activities. However, chymotrypsin-, trypsin- and caspase-like activities were similar in BMDM of both genotypes, as measured by using short fluorogenic peptides in BMDM whole cell lysates (Fig. 3C).

## Discussion

The major finding of the current study is that the deficiency of the IP subunit β5i/LMP7 does not aggravate initiation and progression of atherosclerosis in LDLR^−/−^ mice. This is of particular interest, since β5i/LMP7 expression was suggested to be required for the efficient elimination of damaged proteins after cytokine induced oxidative stress^14^. However, this relationship was questioned by others^15^, leading to a debate on the role of β5i/LMP7 in the pathogenesis of inflammatory disorders^19^. The present study contributes to this debate, as our observations do not point to an impact of β5i/LMP7-deficiency on atherosclerosis, which is considered a chronic inflammatory vascular disease^20^.

It has been hypothesized that the UPS is dysregulated in atherosclerosis^20,21^ and that the accumulation of dysfunctional proteins is associated with cell death within atherosclerotic lesions^3^. Given the proposed importance of β5i/LMP7 for the removal of damaged proteins^14^, aggravation of atherosclerosis in β5i/LMP7-deficient mice would have been conceivable. However, we did not detect augmented ubiquitin accumulation in plaques of β5i/LMP7-deficient mice. In line with previous reports^3,20^, ubiquitin enrichment was associated with macrophages, but was equally present in the plaques of both genotypes (considering the limited sample size). Furthermore, the similar extent of necrotic cores in atherosclerotic lesions of both genotypes does not indicate increased cell death in β5i/LMP7-deficient mice. Finally, atherosclerotic plaque size was not affected by β5i/LMP7-deficiency for early or advanced stage atherosclerosis. Thus, these findings do not favor the perception that β5i/LMP7-containing proteasomes have a higher capacity to degrade polyubiquitinated proteins compared to standard proteasomes.

Recently, beneficial effects of β5i/LMP7-deficiency on obesity and glucose homeostasis have been described in the presence of mild hypercholesteremia^22^. β5i/LMP7-deficient mice fed a high fat diet showed an improved glucose intolerance, lower triglyceride levels and a decreased weight gain when compared to C57BL/6 wildtype mice^22^. Such metabolic alterations are potentially important to atherogenesis. However, in our study β5i/LMP7-deficiency on a LDLR^−/−^ background did not lead to significant alterations of glucose levels. Also, weight gain during short and long term high-fat WD was not affected by β5i/LMP7-deficiency. We observed higher triglyceride levels in β5i/LMP7-deficient mice fed a WD over 6 weeks; triglycerides are associated with risk of cardiovascular disease in humans. However, atherosclerosis outcome was not affected by β5i/LMP7-deficiency in our model. We cannot exclude the possibility that a potential impact of elevated triglycerides on atherogenesis has been outweighed by other (yet unidentified) effects of β5i/LMP7-deficiency. Thus, further in depth characterization of lipoprotein forms in β5i/LMP7-deficient models should be performed in future studies.

Macrophages play a key role in atherosclerotic lesion formation, progression and plaque destabilization. Depending on the cytokine milieu within atherosclerotic lesions macrophages are polarized towards distinct subsets, which critically affect plaque development^18^. It was recently reported that β5i/LMP7-deficiency and selective inhibition of β5i/LMP7 promotes M2 polarization of alveolar macrophages^23^. Our data derived from BMDM do not suggest an impact of β5i/LMP7 on M1 and M2 polarization as well as on macrophage survival under oxidative stress.

In line with previous reports, we observed that β5i/LMP7-deficiency leads to the formation of proteasome subtypes with β5i/LMP7 being replaced by its corresponding standard subunit β5^24,25^. Structure and function of various proteasome subpopulations and -types have been investigated in different species and organs^24,26–29^. Studies comparing the enzymatic activities of different isolated proteasome subtypes *in vitro* using fluorogenic peptide substrates revealed varying activities and differential cleavage characteristics depending on the proteasomal subunit composition^5,25,27^. However, despite differences in subunit composition we found similar proteolytic activities when measured in BMDM whole cell lysates of both genotypes using short fluorogenic peptides. In addition, the detection of similar amounts of accumulated polyubiquitinated proteins in hydrogen peroxide stimulated BMDM of both genotypes further confirmed an equal capacity for the removal of damaged proteins under oxidative stress; taken together, these observations are in accordance with the findings of Nathan *et al*.^15^.

In conclusion, deficiency of IP subunit β5i/LMP7 does not alter initiation and progression of atherosclerosis in LDLR^−/−^ mice. Our data indicate that protein homeostasis in atherosclerosis is not disturbed by deficiency of β5i/LMP7.

## Methods

### Materials

Unless otherwise specified, all reagents and media were purchased from Sigma Chemicals, Germany. Bortezomib was kindly provided by Millenium Pharmaceuticals.

### Animal experiments

Animal experiments were approved by the local authority (Landesamt für Gesundheit und Soziales, Berlin, Germany) and were performed according to institutional guidelines.

Low-density lipoprotein receptor-deficient (LDLR^−/−^) mice (B6.129S7-*Ldlr* ^*tm1Her*^/J; JAX Mice, Boston) and β5i/LMP7^−/−^ mice (kindly provided by Antje Voigt, Department of Biochemistry, Charité-Universitätsmedizin Berlin, Germany) were crossed to generate double heterozygous mice. Both, LDLR^−/−^ and β5i/LMP7^−/−^ mice were originally backcrossed at least 10 times to C57BL/6 mice. The resulting F1 generation was crossed and the offsprings genotyped for LDLR and β5i/LMP7. Male mice homozygous for LDLR-deficiency (LDLR^−/−^) or homozygous for LDLR-deficiency and β5i/LMP7-deficiency (LDLR^−/−^LMP7^−/−^) were fed a high fat diet containing 21% butterfat, 17% casein, and 0.21% cholesterol (Western-type diet, Ssniff, Soest, Germany) for 6 or 24 weeks *ad libitum* beginning from 10 weeks of age. Mice of both genotypes fed a low-fat control diet (CD) served as additional controls. General condition and body weight were monitored regularly.

For harvesting, mice were fasted for two hours, anesthetized with isoflurane- and euthanized by blood withdrawal. After perfusion with PBS, heart and aorta were dissected under a stereomicroscope (Leica), shock-frozen in liquid nitrogen and stored at −80 °C or fixed in formalin. Livers and spleens were collected, shock-frozen and stored at −80 °C.

### Echocardiography

Transthoracic Doppler echocardiography was performed in mice anesthetized with isoflurane (2%) with the use of the high-resolution imaging system Vevo 770 (VisualSonics). M-mode tracings were recorded from the short-axis view of the left ventricle (LV) at the level of the papillary muscles with two-dimensional image guidance through the anterior and posterior walls. Left ventricular internal dimensions were measured at the end of diastole (LVIDd) and systole (LVIDs). Ejection fraction (EF) and fractional shortening (FS) were calculated from linear measurements of LVIDd and LVIDs.

### Measurement of serum glucose and lipids

Serum glucose levels were measured using a Fuji DRI-CHEM NX500 system (Fujifilm). Serum total cholesterol and triglyceride concentrations were measured by colorimetric enzymatic assay (CHOL-PAP, and TG GPO-PAP, Roche-Diagnostics). High-density lipoprotein cholesterol concentration was separated by the phosphotungstate-magnesium precipitation technique. Briefly, 100 μl of serum were supplemented with 10 μl sodium phosphotungstate solution (40 g of phosphotungstic acid per liter in 160 mM NaOH) and 5 μl 1 M MgCl2. After mixing and incubation for 2 hours; samples were centrifuged (1500 g, 30 minutes, 4 °C) and cholesterol was determined in the supernatant.

### Measurement of thiobarbituric-acid reactive substances (TBARS)

Lipid hydroperoxides were determined in mouse serum by spectrophotometric measurement of formation of thiobarbituric-acid reactive substances (TBARS), as described previously^30^.

### Staining and analysis of atherosclerotic lesions

The dissected aorta was fixed in formalin overnight, opened longitudinally under a stereomicroscope, and pinned flat on a silicon gel. For *en face* aortic lesion analysis, pinned aortae were stained with Oil Red O (Sigma-Aldrich) and digitally photographed at standardized magnification and illumination. Total aortic area and atherosclerotic lesion area were determined using ImageJ software. Atherosclerotic lesion area was calculated as percentage of total *en face* aortic area.

Cross sections of formalin-fixed, paraffin-embedded brachiocephalic arteries (BCA, *truncus brachiocephalicus*) were stained with the original Movat pentachrome according to the manufacturers’ protocol (Morphisto). Frozen acetone-fixed cross sections (5 µm) of aortic roots were stained with Oil Red O (counterstained with hematoxylin) or with Movat pentachrome. Sections were digitally photographed under standardized conditions using Zeiss AxioCam MrC and analyzed using Zeiss AxioVision software. Absolute plaque size (in µm^2^) was determined as well as absolute cell free necrotic core area. Plaque macrophages and ubiquitinated proteins were detected in cross sections by immunohistochemistry using anti-Mac-2 antibody (Cedarlane Laboratories), or anti-FK2 ubiquitin monoclonal antibody (Enzo Life Sciences), respectively. Biotin-conjugated goat anti-mouse IgG (Jackson Immunoresearch) was used as secondary antibody, followed by 10 minutes incubation with streptavidin-peroxidase (Zymed) and visualization with diaminobenzidine (DAB, Zymed). For macrophage content Mac-2 positive plaque area was determined (in µm^2^). After ubiquitin staining, sections were stained with 4’, 6-diamidino-2-phenylindole (DAPI) to visualize cell nuclei. Images of 3–4 regions of interest per plaque were analyzed and the mean percentage of cells with strong ubiquitin signal was calculated. CD4 immunofluorescence for analysis of plaque T cells was performed using rat anti-mouse anti-CD4 (BD Biosciences) and Cy3-conjugated donkey anti-rat IgG (Jackson Immunoresearch) as secondary antibody. Nuclei were stained with DAPI. Sections were photographed with a Zeiss Axioplan-2 microscope and HrC AxioCam, plaque area was measured using AxioVison software, CD4 cell numbers per plaque area were counted.

### Cell Culture and treatments

Primary bone marrow derived macrophages (BMDM) were recovered from bone marrow suspensions of tibia and femur of LDLR^−/−^LMP7^−/−^ and LDLR^−/−^ littermate mice as described previously^31^. For differentiation in macrophages, cells were incubated in RPMI-1640 (Gibco Life Technologies) containing 10% fetal calf serum, 1% penicillin/streptomycin, and 10% of L929-conditioned RPMI (as a source of Macrophage Colony Stimulating Factor, M-CSF) for 7 days. Macrophage differentiation was verified by flow cytometry via staining for CD45 (APC, BioLegend), F4/80 (PE, Abcam) and C11b (V450, BD Horizon) on day 7. Mouse ear fibroblasts have been isolated and cultivated as described previously^32^.

For individual experiments, BMDM or fibroblasts were treated with 100 U/ml IFN-γ (recombinant, Biomol) for varying times or with medium containing varying concentrations of hydrogen peroxide (H_2_O_2_; Sigma) at 37 °C for 60 minutes. For survival analyses, H_2_O_2_-treated BMDM were harvested by scraping, and counted in a hemocytometer. Numbers of survived cells were expressed as percentage of cell count of the corresponding untreated control. For polarization of macrophages into the M1 and M2 state, BMDMs were incubated with 100 U/ml IFN-γ or 5 U/ml IL-4 (Promokine) at 37 °C for 6 hours, respectively.

### Quantitative real-time RT-PCR

RNA was isolated using RNeasy kit (Qiagen) according to the manufacturer’s instructions. 500 ng of total RNA was reversed-transcribed with random hexamers (TIB Molbiol) using Reverse Transcriptase Kit (Invitrogen). TaqMan assays (Applied Biosystems) were used to quantify the expression of tumor necrosis factor-α (TNF-α; Mm00443260_g1), monocyte chemoattractant protein-1 (MCP-1; Mm00441242_m1), arginase-1 (Mm00475988_m1), and proteasome subunits β1 (Mm01245590_g1), β2 (Mm00650844_g1), β5 (Mm01615821_g1), β1i/LMP2 (Mm00479004_m1), β5i/LMP7 (Mm01278979_m1), β2i/MECL1 (Mm00479052_g1) using the 7300 Real Time PCR System (Applied Biosystems). RPL19 (Mm02601633_g1) was used as housekeeping gene. Expression of the target gene relative to housekeeping gene expression was calculated as the difference between the threshold values for the two genes.

### Measurement of superoxide production

Superoxide production was measured using electron paramagnetic resonance (EPR) technique as described previously^33,34^. Briefly, BMDM were washed twice in Krebs HEPES buffer, pH 7.35 (99 mM NaCl, 4.69 mM KCl, 25 mM NaHCO_3_, 1.03 mM KH_2_PO_4_, 5.6 mM d-glucose, 20 mM Na-HEPES, 2.5 mM CaCl_2_, 1.2 mM MgSO_4_) and incubated in Krebs-HEPES buffer supplemented with 25 µM desferoxamine, 5 µM diethyldithiocarbamate and 100 µM of the spintrap 1-hydroxy-3-methoxycarbonyl-2,2,5,5-tetramethylpyrrolidine (CMH; Noxygen) for 20 minutes on ice followed by snap freezing in liquid nitrogen. Superoxide generation was analyzed at 37 °C for 10 minutes with 20 scannes in an Escan EPR spectrometer with temperature control (Noxygen) with the following starting parameters: microwave power 23.89 mW; center field 3459–3466 G; steep width 10 G, frequency 9.7690 GHz and modulation amplitude 2.93 G. Superoxide generation rate was calculated using linear regression and normalized to the protein content of cells.

### Western blot analysis

BMDM and spleen specimen were lysed in extraction buffer containing (in mmol/L): Tris/HCl 50 (pH 7.4), KCl 154, glucose 5, EDTA 0.5, Complete protease inhibitor, and 1% Triton X-100. We isolated protein from livers and spleens separately for each animal, and used equal amounts of protein per animal to prepare one pooled sample per group. Total protein (10 μg per lane) was subjected to SDS-PAGE and membranes were probed with the respective antibodies: anti-Ubiquitin (DAKO), anti-β5 (Abcam), anti-β1 (Abcam), anti-β2 (Abcam), anti-β1i/LMP2 (Abcam), anti-β5i/LMP7 (Epitomics), anti-β2i/MECL-1, anti-α4 (kindly provided by the Department of Biochemistry, Charité-Universitätsmedizin Berlin, Germany). After probing with the respective secondary antibodies, bands were visualized using ECL detection system (Amersham) and chemiluminescence system Fusion Solo (Vilber Lourmat). Amidoblack staining or probing with antibodies against GAPDH (Santa Cruz) or β-actin (MAB150, Millipore) served as controls for equal protein loading. Densitometry was performed using ImageJ software.

### Proteasomal activity assay and assessment of proteasome subunit composition

Proteasome chymotrypsin-like (ChTL), trypsin-like (TL) and caspase-like (CaspL) activities of BMDM lysates were determined fluorometrically in a spectramax GEMINI-EM (Molecular Devices) by using synthetic peptides linked to the fluorophor 7-amino-4-methylcoumarin (AMC). ChTL activity was measured by SLLVY-AMC, TL by BzVGR-AMC and CaspL activity by ZLLE-AMC hydrolysis with 360-nm excitation and 460-nm emission wavelengths. BMDM were lysed under hypotonic conditions with three cycles of thawing and freezing in liquid nitrogen. Lysates were centrifuged and the protein content of the supernatant was estimated using the BCA Protein Assay kit (Pierce).

For activity assays, 10 µg of protein were incubated at 37 °C for 30 minutes in incubation buffer containing an ATP-regenerating system (225 mM Tris–HCl, pH 8.2, 45 mM KCl, 7.5 mM Mg(CH_3_COO)_2_, 7.5 mM MgCl_2_, 1.1 mM dithiothreitol, 6 mM ATP, 5 mM phosphocreatine, 0.2 unit of phosphocreatinekinase) and 0.2 mM of the appropriate fluorogenic substrate. Proteasomal activity was determined by calculating the difference of AMC formation in the absence and presence of 10 µM MG262 and 20 µM epoxomicin and the results were expressed as AMC formation (relative fluorescence units).

For assessment of proteasome subunit composition, lysates were separated on non-denaturing 3–8% Tris-acetate polyacrylamide gels (Life Technologies) and 26 S proteasomes were localized after overlay with 100 µM SLLVY-AMC and 5 mM ATP at 37 °C for 30 minutes using an UV transilluminator. 26 S proteasome containing gel slices were extracted by centrifugation through Amicon Ultra-2 Centrifugal Filter Devices (Millipore) in 120 mM Tris HCl pH 6.8, 0.2 M DTT, 4% SDS, 0.002% bromophenol blue and 20% glycine at 4 °C. Protein extracts were separated under denaturing conditions on 10% polyacrylamide gels and proteasome subunits were detected by Western blot with the respective specific antibodies. At first, a Western blot with anti-α4 antibody was performed and signal intensities were used for the adjustment of equal proteasomal protein loading in subsequent Western blots.

### Statistical analysis

Data were analyzed using GraphPad Prism software version 7. Data variability about the mean is generally expressed as standard error of the mean (SEM), except where otherwise indicated. Continuous variables were tested for normal distribution by Shapiro-Wilk test and skewness and for equality of variances by F test. Unpaired Student’s *t*-test (Welch test for unequal variances) was used for normally distributed variables. A two-way analysis of variance (ANOVA) followed by Sidak’s multiple comparisons test was used to analyze experiments with multiple independent factors across genotypes. Mann-Whitney *U* test or Kruskal-Wallis test with post hoc comparison by Dunn’s multiple comparison test was used for non-normally distributed variables. p < 0.05 was considered statistically significant.

## Electronic supplementary material

Supplementary Tables and Figures

Full-length Western Blots
