# Supplementary material for: Immunoproteasome subunit ß5i/LMP7-deficiency in atherosclerosis
Source: Sci Rep. 2017 Oct 17;7:13342. doi: 10.1038/s41598-017-13592-w (PMC5645401; doi:10.1038/s41598-017-13592-w)
Supplement: Supplementary file 1 — Supplementary Tables and Figures [file 41598_2017_13592_MOESM1_ESM.pdf]

Supplementary Information

Immunoproteasome subunit ß5i/LMP7-deficiency in atherosclerosis

Bernd Hewing[1,2,7], Antje Ludwig[1,2], Cristian Dan[1,2], Max Pötzsch[1,2], Carmen Hannemann[1,2], Andreas Petry[3,4], Dilyara Lauer[5], Agnes Görlach[3,4], Elena Kaschina[5], Dominik N. Müller[2,6,7,8] Gert Baumann[1], Verena Stangl[1,2], Karl Stangl[1,2], Nicola Wilck[1,2,6,8]

[1]Medizinische Klinik m.S. Kardiologie und Angiologie, Charité-Universitätsmedizin Berlin, Campus Mitte, Berlin, Germany
[2]DZHK (German Center for Cardiovascular Research), partner site Berlin, Germany
[3]Experimental and Molecular Pediatric Cardiology, German Heart Center Munich, Technical University Munich, Munich, Germany
[4]DZHK (German Center for Cardiovascular Research), partner site Munich, Germany
[5]Institute of Pharmacology, Center for Cardiovascular Research, Charité-Universitätsmedizin Berlin, Berlin, Germany
[6]Experimental and Clinical Research Center, a joint cooperation of Max Delbrück Center for Molecular Medicine and Charité Medical Faculty, Berlin, Germany
[7]Berlin Institute of Health (BIH), Berlin, Germany
[8]Max Delbrück Center for Molecular Medicine in the Helmholtz Association, Berlin, Germany

Supplement tables

Supplement table S1: Body weight and lipid levels

	6 weeks control diet			24 weeks control diet		
	**LDLR$^{-/-}$**	**LDLR$^{-/-}$LMP7$^{-/-}$**	**p value**	**LDLR$^{-/-}$**	**LDLR$^{-/-}$LMP7$^{-/-}$**	**p value**
**Body weight, g**	26.6 ± 2.3	26.7 ± 1.7	0.967	30.7 ± 2.0	27.9 ± 3.5	**0.034**
**Total cholesterol, mg/dl**	286.7 ± 33.3	284.7 ± 54.7	0.920	290.2 ± 22.1	276.4 ± 31.1	0.245
**HDL-C, mg/dl**	90.9 ± 19.2	96.3 ± 7.4	0.4	128.1 ± 12.7	124.8 ± 19.8	0.818
**Non-HDL-C, mg/dl**	195.9 ± 19.2	188.4 ± 56.9	0.695	162.1 ± 53.4	151.6 ± 31.9	0.582
**Triglycerides, mg/dl**	164.9 ± 38.5	121.6 ± 48.2	**0.034**	94.4 ± 13.5	81.2 ± 20.0	0.083

n=11 per group; mean ± SD. Statistical analysis using unpaired *t*-test (Welch test for unequal variances). HDL-C, high-density lipoprotein cholesterol.

Supplement Table S2: Cardiac dimensions and function

	6 weeks control diet			6 weeks WD		
	**LDLR$^{-/-}$**	**LDLR$^{-/-}$LMP7$^{-/-}$**	**p value**	**LDLR$^{-/-}$**	**LDLR$^{-/-}$LMP7$^{-/-}$**	**p value**
**n**	**5**	**5**		**4**	**3**	
**LVIDd, mm**	4.0 ± 0.3	3.8 ± 0.3	0.256	3.9 ± 0.1	3.7 ± 0.2	0.286
**LVIDs, mm**	2.7 ± 0.4	2.6 ± 0.2	0.503	2.5 ± 0.1	2.5 ± 0.2	0.8
**LVEF, %**	60.9 ± 7.5	61.0 ± 3.1	0.98	65.1 ± 1.9	62.4 ± 4.3	0.4
**FS, %**	32.4 ± 5.2	32.3 ± 2.2	0.948	35.2 ± 1.4	33.1 ± 3.2	0.4
**Stroke volume, µl**	42.0 ± 4.2	37.3 ± 6.4	0.203	41.2 ± 3.3	36.3 ± 6.3	0.230

Mean ± SD. Statistical analysis using unpaired *t*-test or Mann-Whitney *U* test. WD, Western-type diet; LVIDd, end-diastolic left ventricular inner dimension; LVIDs, systolic left ventricular inner dimension; FS, fractional shortening; LVEF, left ventricular ejection fraction.

Supplement Figure S1

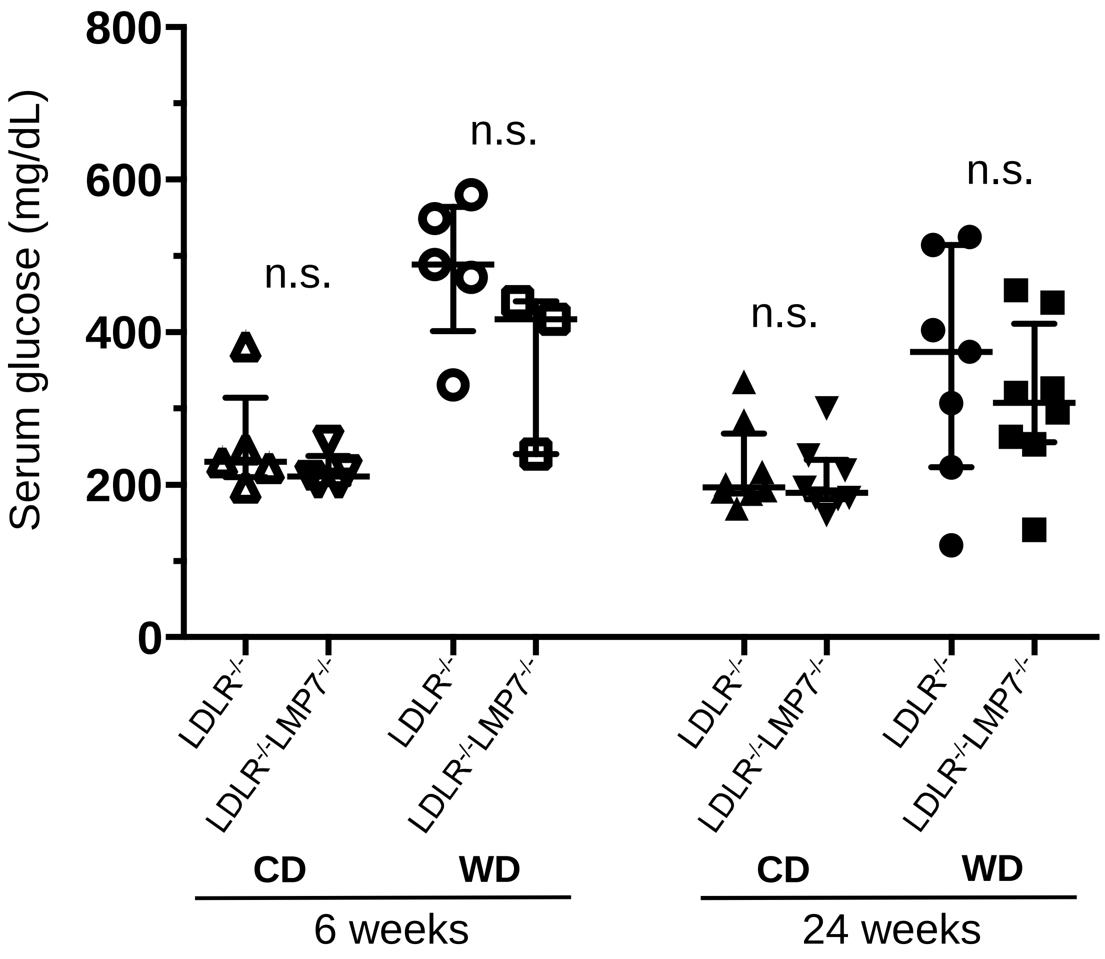

Supplement Figure S1. Serum glucose levels. $LDLR^{-/-}LMP7^{-/-}$ and $LDLR^{-/-}$ mice were fed a control (CD) or a high-fat Western-type diet (WD) for either 6 or 24 weeks. Data presented as mean ± SEM. n.s.= statistically non-significant using unpaired *t*-test or Mann-Whitney *U* test.

Supplement Figure S2

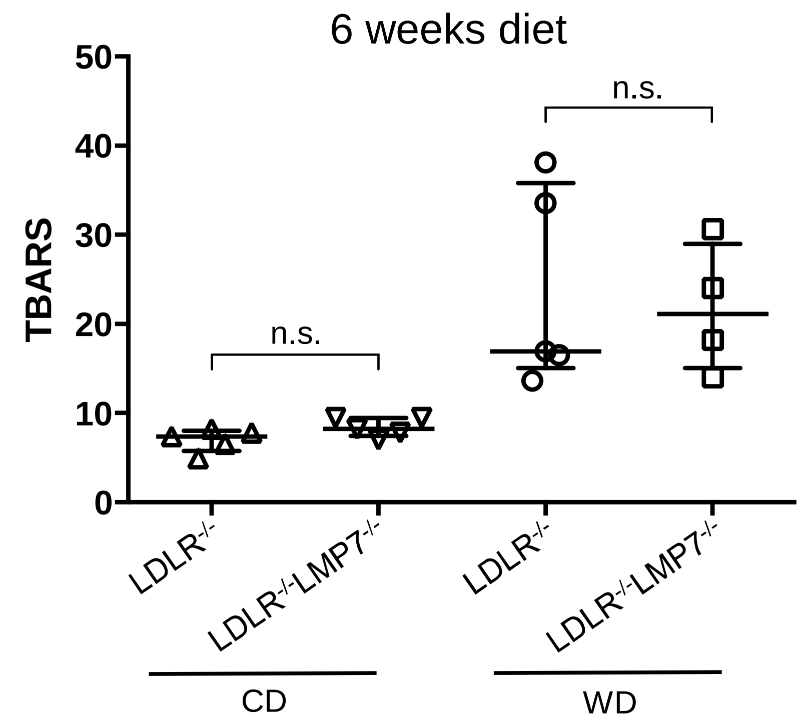

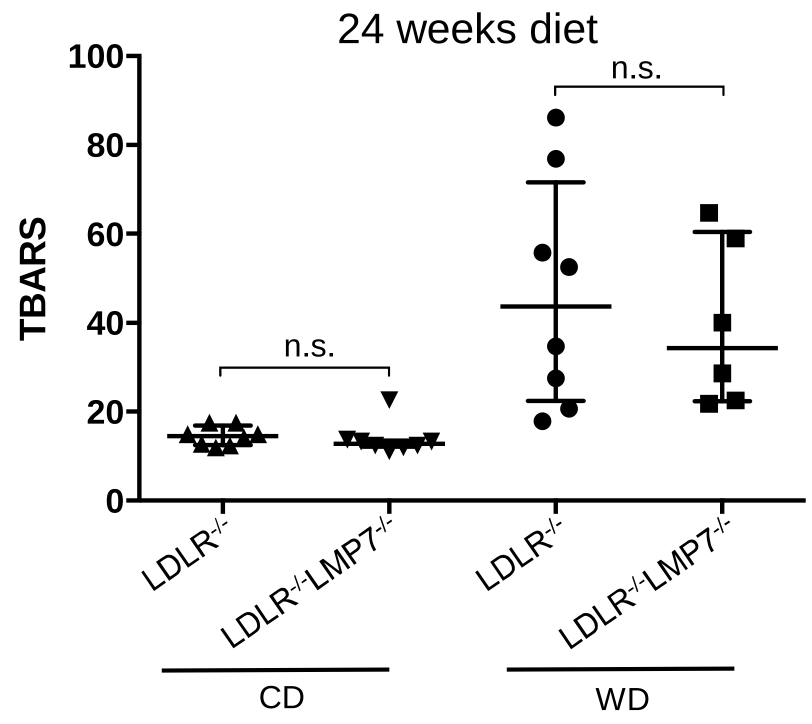

Supplement Figure S2. Serum levels of thiobarbituric acid reactive substances (TBARS). LDLR$^{-/-}$LMP7$^{-/-}$ and LDLR$^{-/-}$ mice were fed a control (CD) or a high-fat Western-type diet (WD) for either 6 or 24 weeks. Data are presented as individual values with median and interquartile ranges indicated. All comparisons between CD and WD fed mice were statistically significant ($p<0.05$). n.s.= statistically not significant using unpaired *t*-test or Mann-Whitney *U* test.

Supplement Figure S3

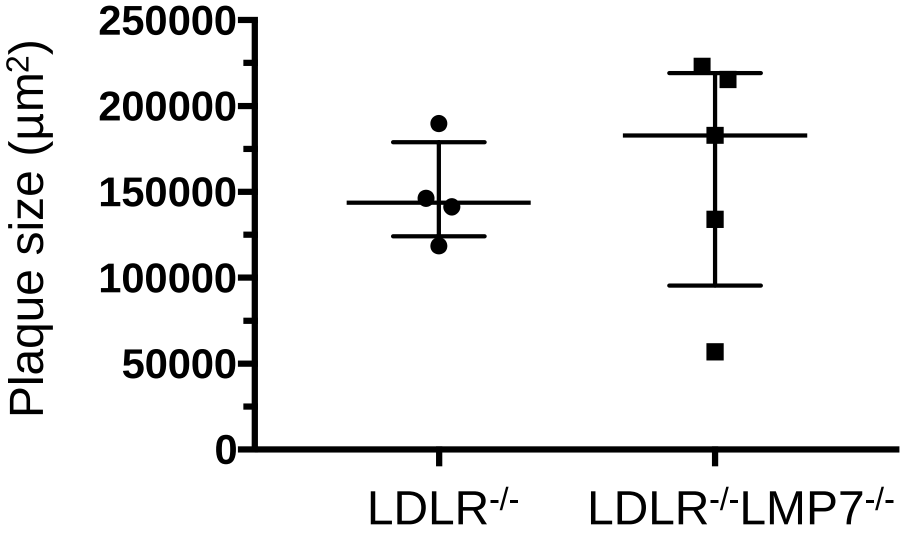

Supplement Figure S3. Plaque size in the *truncus brachiocephalicus*. LDLR$^{-/-}$ LMP7$^{-/-}$ and LDLR$^{-/-}$ mice were fed a high-fat Western-type diet for 24 weeks to induce advanced stage atherosclerosis. Data are presented as individual values with median and interquartile ranges indicated. n.s. = statistically non-significant using Mann-Whitney *U* test.

Supplement Figure S4

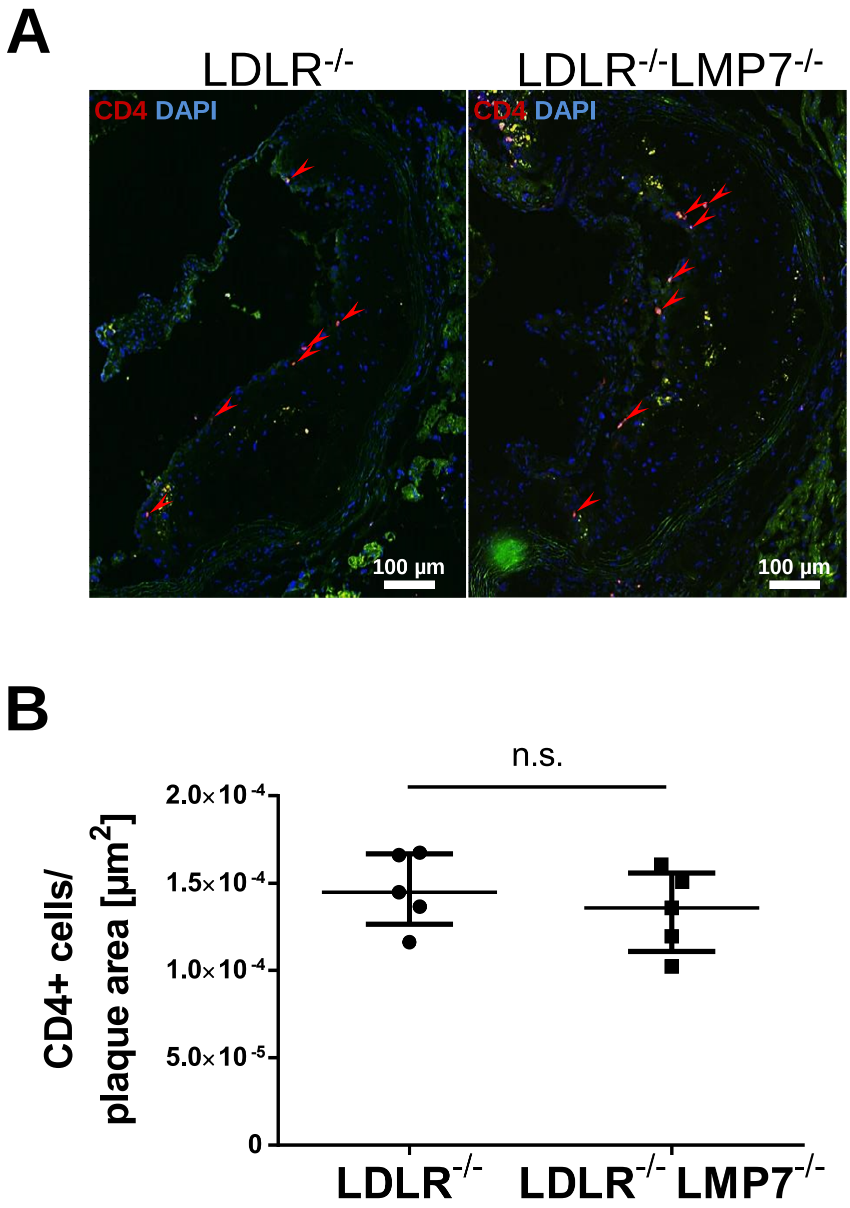

Supplement Figure S4: T cells (CD4) in atherosclerotic plaques of the aortic root. Aortic root cryosections from LDLR$^{-/-}$ and LDLR$^{-/-}$LMP7$^{-/-}$ mice fed a Western-type diet for 24 weeks were analyzed for the number of CD4-positive cells per plaque area. **A** Representative merged images showing anti-CD4 immunofluorescence in red (red arrowheads). Nuclei were stained with DAPI (blue). Autofluorescence in green. **B** Quantification of CD4-positive cells per plaque area. Data presented as individual values with median and interquartile ranges indicated. n=5 per group. n.s.= statistically non-significant using unpaired *t*-test.

Supplement Figure S5

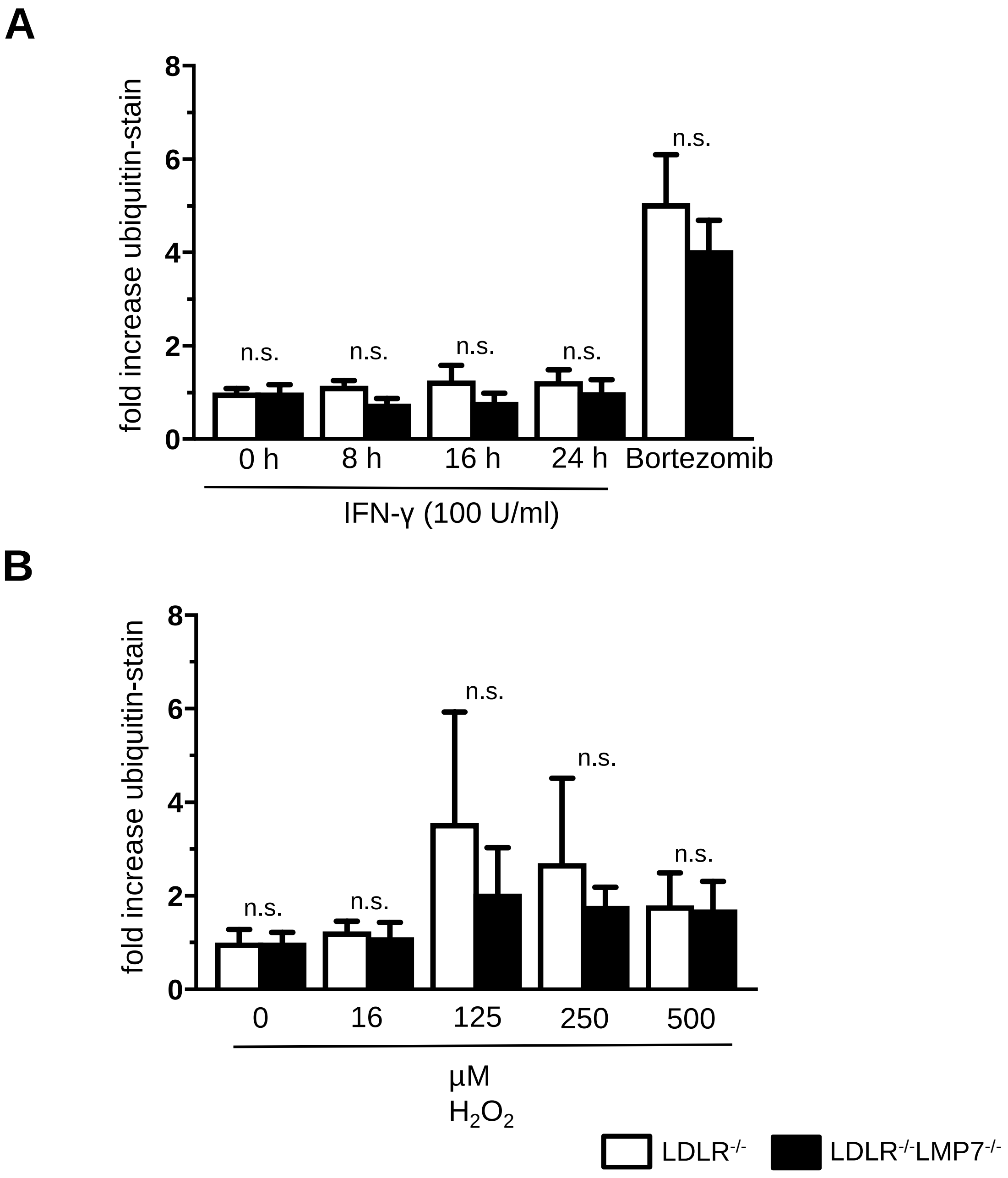

Supplement Figure S5. Densitometric analyses of anti-ubiquitin Western blots (Figure 2C and 2D). Bone marrow derived macrophages (BMDM) of $LDLR^{-/-}LMP7^{-/-}$ and $LDLR^{-/-}$ mice: **(A)** lysates of IFN-γ (100 U/ml) treated BMDM; Bortezomib (5 ng/ml) treated BMDM served as positive controls, n = 4 experiments; **(B)** lysates of BMDM after treatment with hydrogen peroxide (H_2O_2) over 1 hour; n = 3 experiments. Data are presented as mean ± SEM; n.s. = statistically non-significant using two-way ANOVA followed by Sidak's multiple comparisons test.

Supplement Figure S6

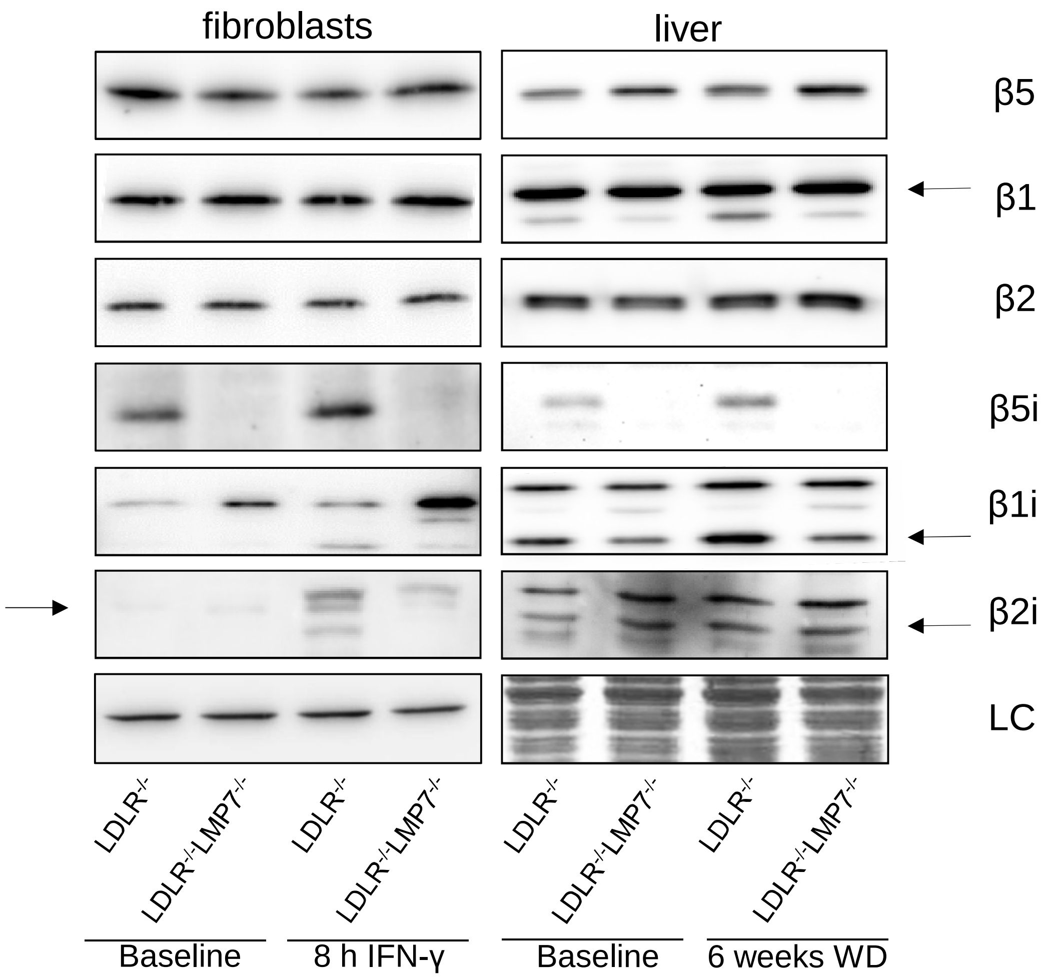

Supplement Figure S6. Impact of β5i/LMP7-deficiency on proteasome composition. Western blot analysis of standard proteasome (β1, β2, and β5) and immunoproteasome (β1i, β2i, and β5i) subunits expression in whole lysates of murine LDLR$^{-/-}$LMP7$^{-/-}$ and LDLR$^{-/-}$ fibroblasts before (baseline) and after treatment with IFN-γ (100 U/ml) for 8 hours (left panel) and of liver lysates of LDLR$^{-/-}$LMP7$^{-/-}$ and LDLR$^{-/-}$ mice. LC indicates loading controls, actin (for fibroblasts) and amidoblack (for liver).

Supplement Figure S7

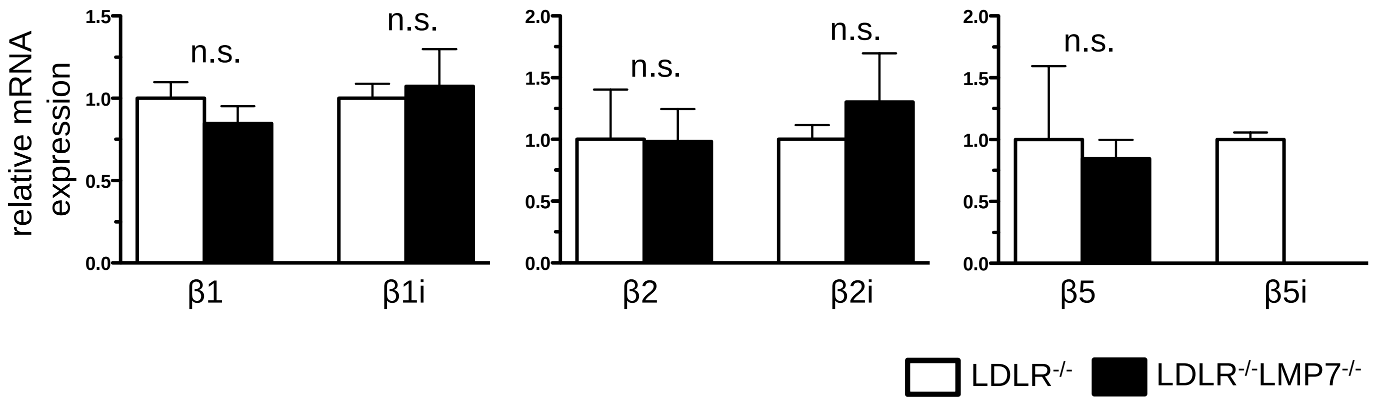

Supplement Figure S7. Impact of β5i/LMP7-deficiency on proteasome composition. mRNA expression determined by quantitative real-time RT-PCR of standard proteasome (β1, β2, and β5) and immunoproteasome (β1i, β2i, and β5i) subunits of murine LDLR$^{-/-}$LMP7$^{-/-}$ and LDLR$^{-/-}$ bone marrow derived macrophages (BMDM); LDLR$^{-/-}$LMP7$^{-/-}$ columns show fold-change in mRNA expression levels relative to expression levels of BMDM of LDLR$^{-/-}$ mice; n = 3 experiments. Data are presented as mean ± SEM; n.s. = statistically non-significant using Mann-Whitney *U* test.
