# Supplementary material for: Immunoproteasome subunit ß5i/LMP7-deficiency in atherosclerosis
Source: Sci Rep. 2017 Oct 17;7:13342. doi: 10.1038/s41598-017-13592-w (PMC5645401; doi:10.1038/s41598-017-13592-w)

Supplementary Information: Full Western Blots

Immunoproteasome subunit ß5i/LMP7-deficiency in atherosclerosis

Bernd Hewing[1,2,7], Antje Ludwig[1,2], Cristian Dan[1,2], Max Pötzsch[1,2], Carmen Hannemann[1,2], Andreas Petry[3,4], Dilyara Lauer[5], Agnes Görlach[3,4], Elena Kaschina[5], Dominik N. Müller[2,6,7,8] Gert Baumann[1], Verena Stangl[1,2], Karl Stangl[1,2], Nicola Wilck[1,2,6,8]

[1]Medizinische Klinik m.S. Kardiologie und Angiologie, Charité-Universitätsmedizin Berlin, Campus Mitte, Berlin, Germany
[2]DZHK (German Center for Cardiovascular Research), partner site Berlin, Germany
[3]Experimental and Molecular Pediatric Cardiology, German Heart Center Munich, Technical University Munich, Munich, Germany
[4]DZHK (German Center for Cardiovascular Research), partner site Munich, Germany
[5]Institute of Pharmacology, Center for Cardiovascular Research, Charité-Universitätsmedizin Berlin, Berlin, Germany
[6]Experimental and Clinical Research Center, a joint cooperation of Max Delbrück Center for Molecular Medicine and Charité Medical Faculty, Berlin, Germany
[7]Berlin Institute of Health (BIH), Berlin, Germany
[8]Max Delbrück Center for Molecular Medicine in the Helmholtz Association, Berlin, Germany

BMDM: β1

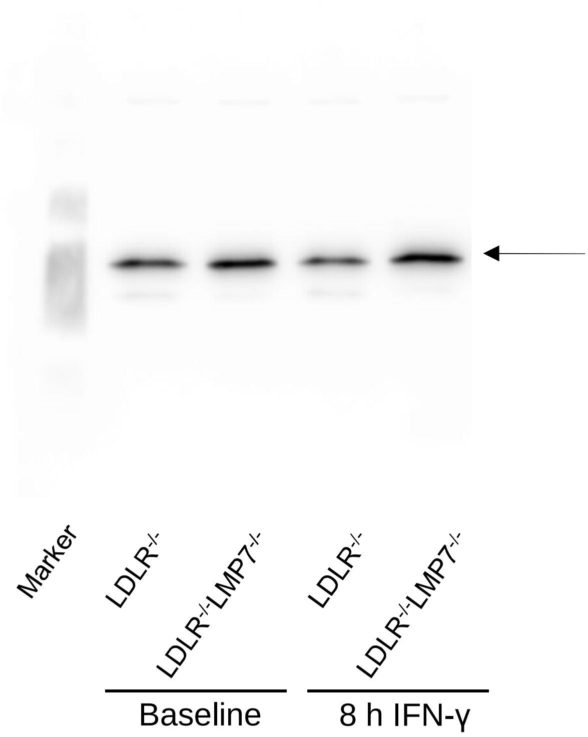

spleen: β1

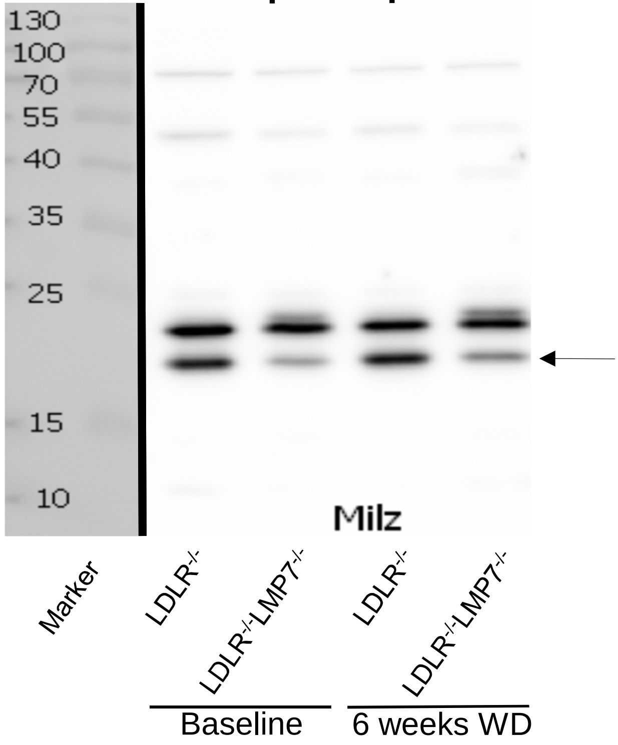

BMDM: β1i

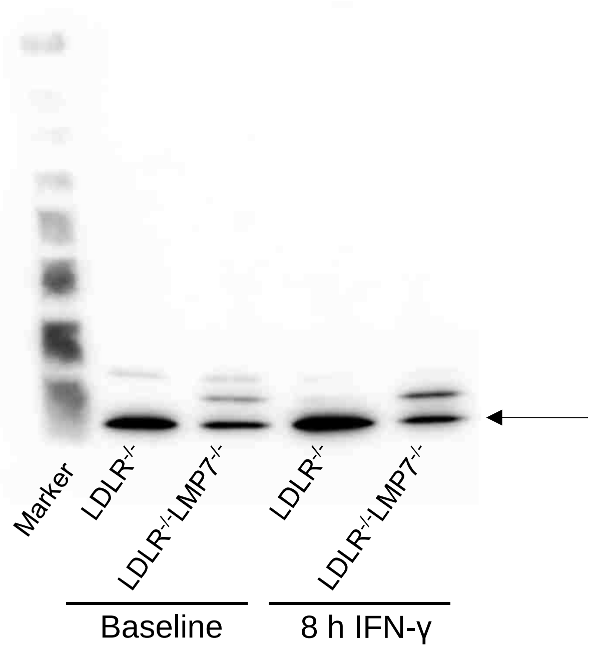

spleen: β1i

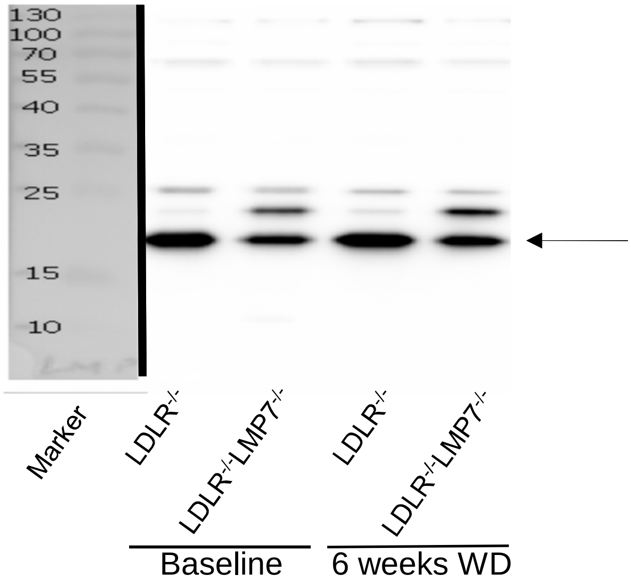

BMDM: β2

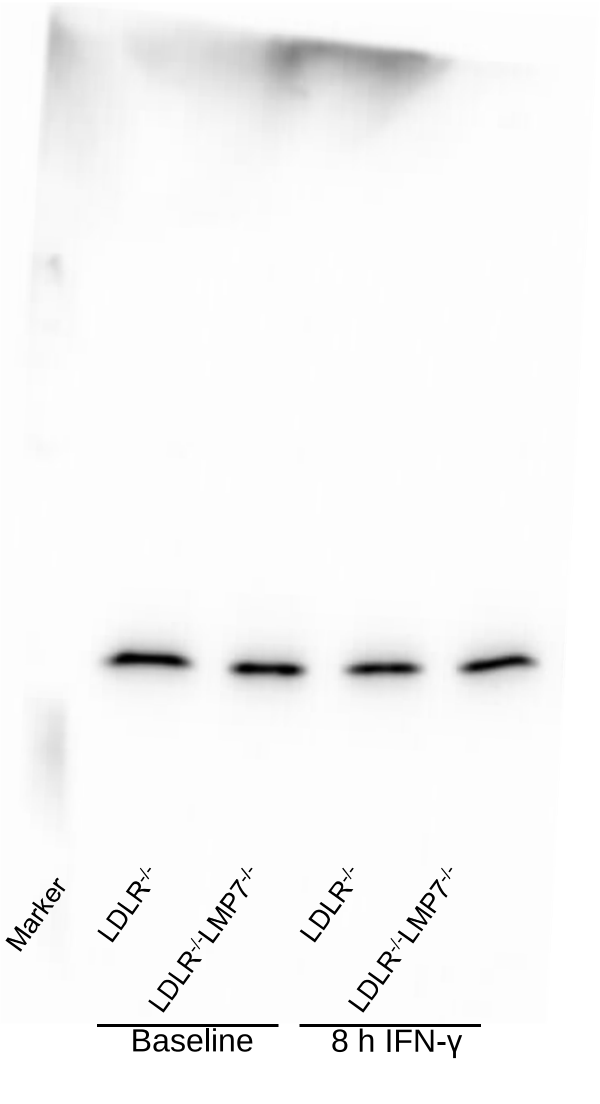

spleen: β2

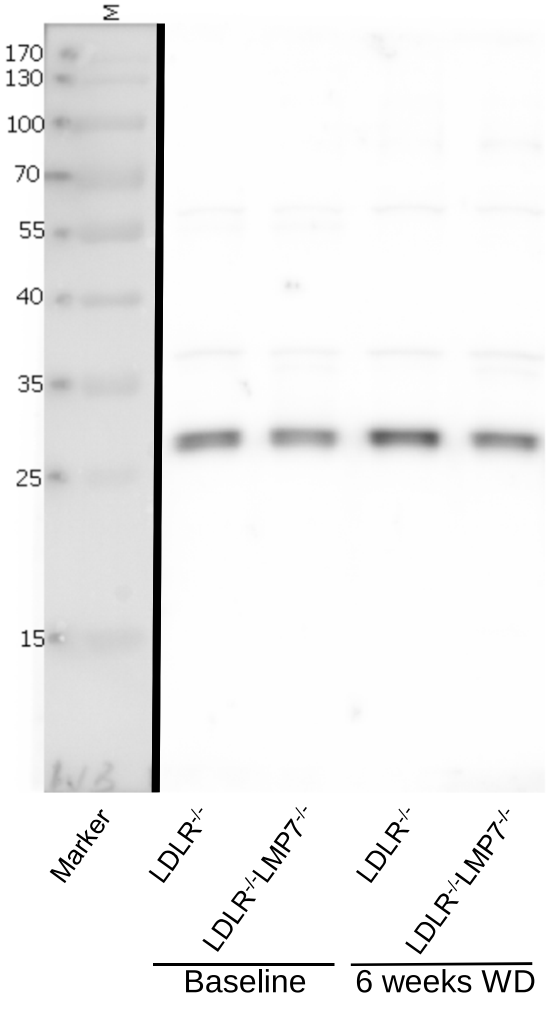

BMDM: β2i

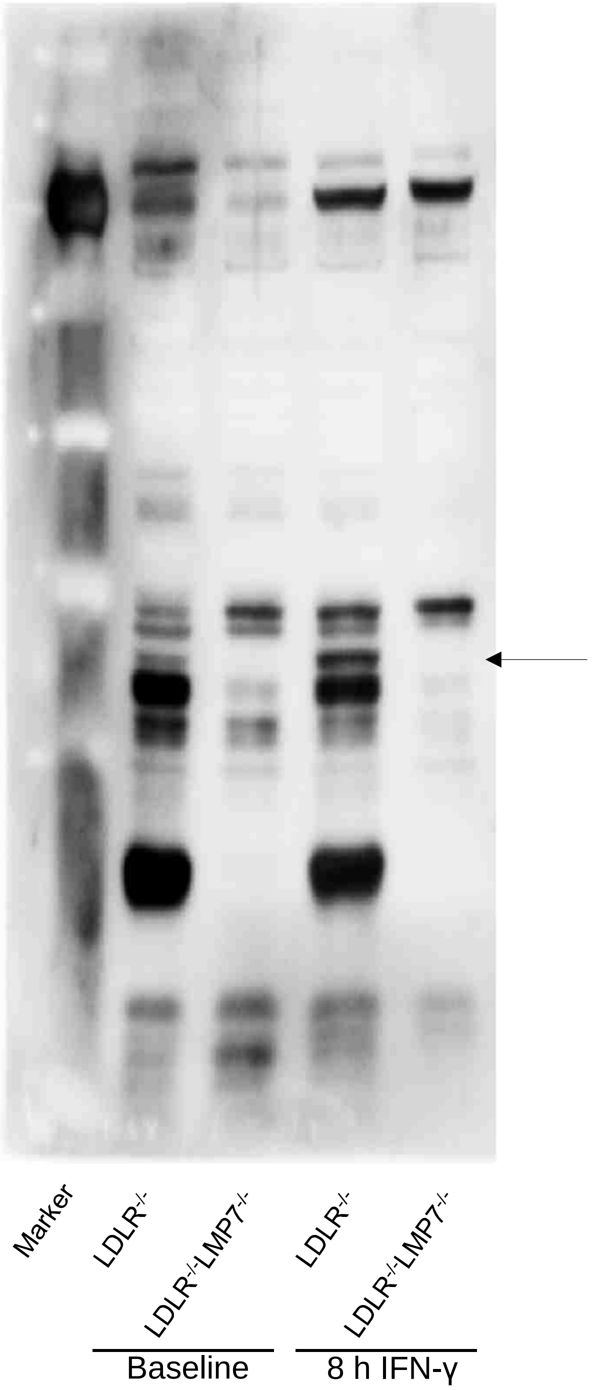

spleen: β2i

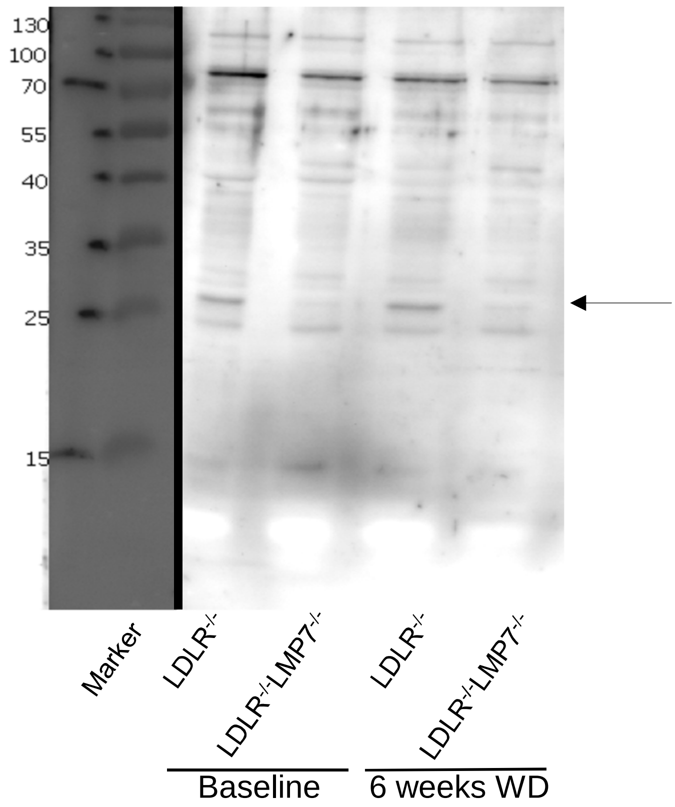

BMDM: β5

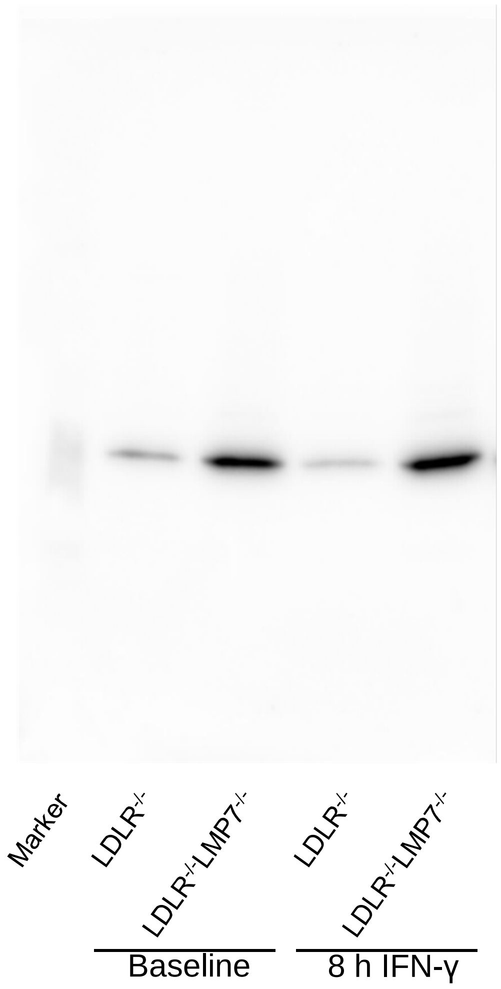

spleen: β5

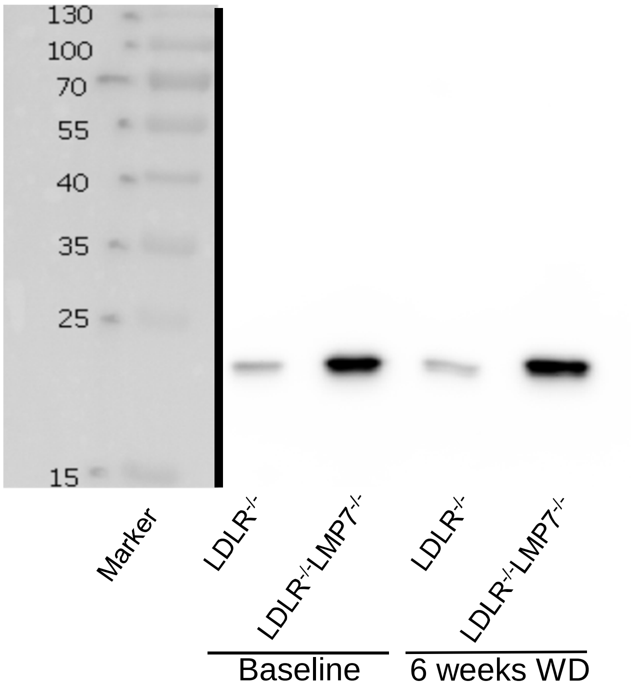

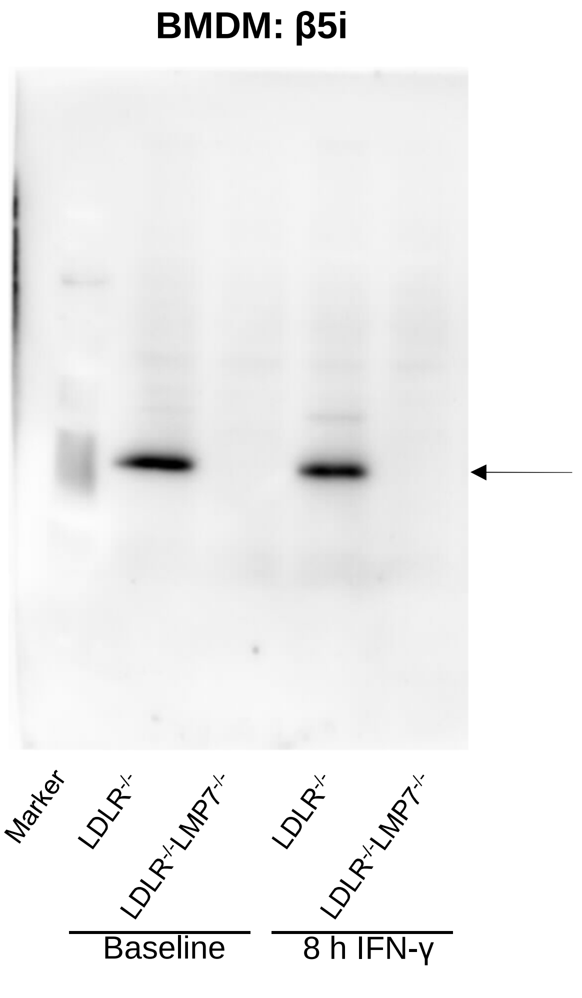
BMDM: β5i
Marker
LDLR-/-
LDLR-/-LMP7-/-
LDLR-/-
LDLR-/-LMP7-/-
Baseline
8 h IFN-γ

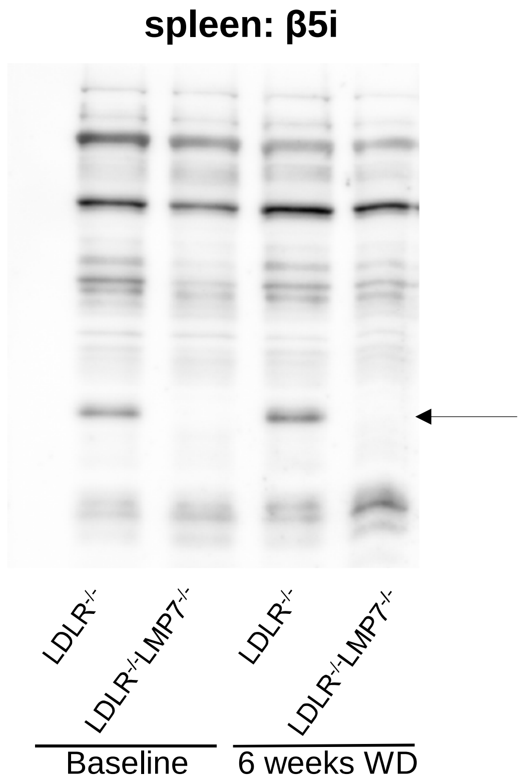
spleen: β5i
LDLR-/-
LDLR-/-LMP7-/-
LDLR-/-
LDLR-/-LMP7-/-
Baseline
6 weeks WD

26S BMDM: α4

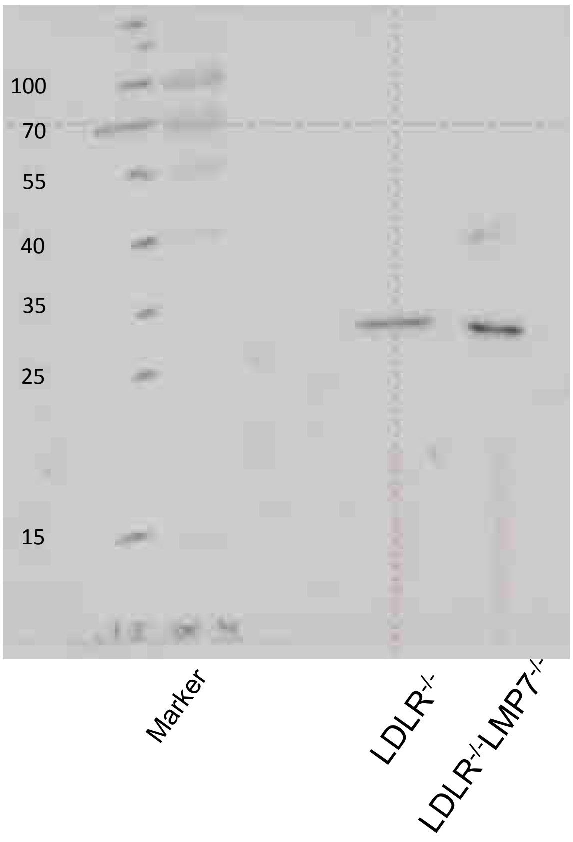

26S spleen: α4

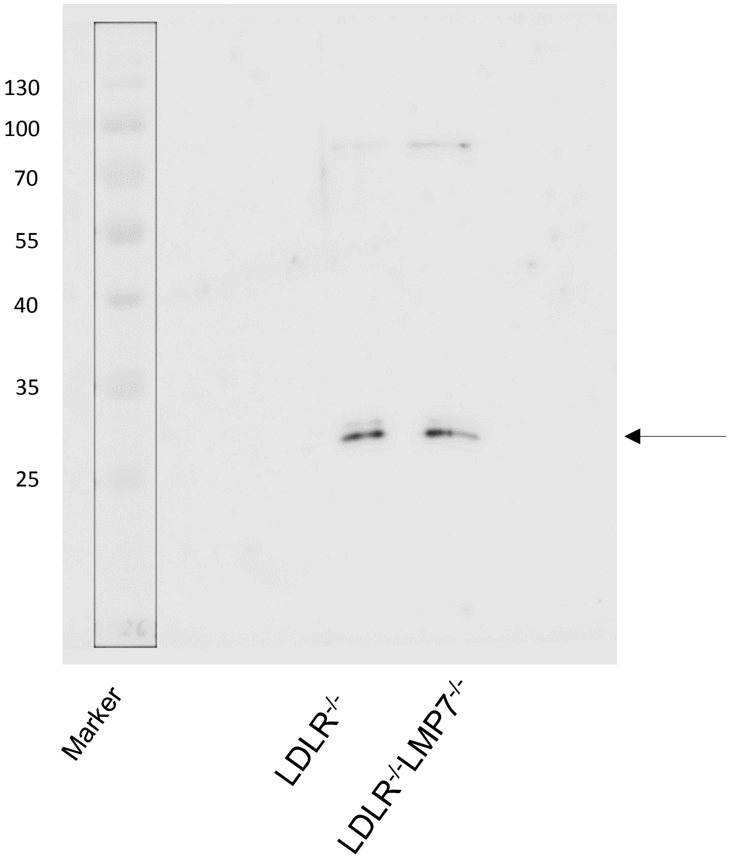

26S BMDM: β5

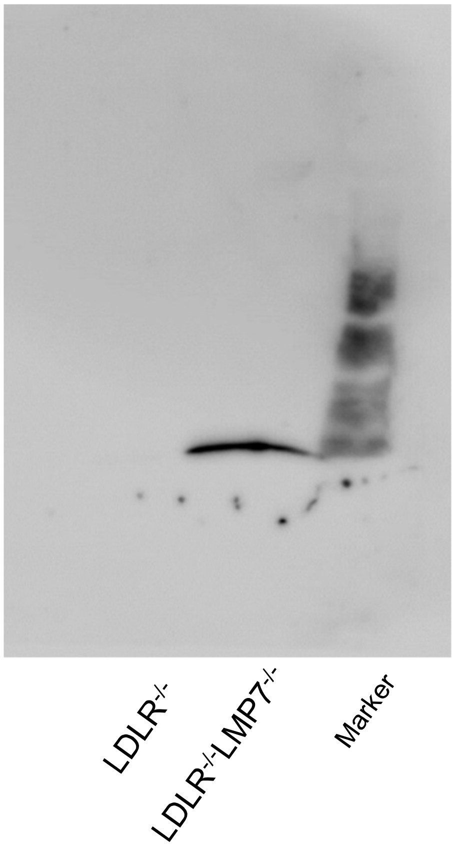

26S spleen: β5

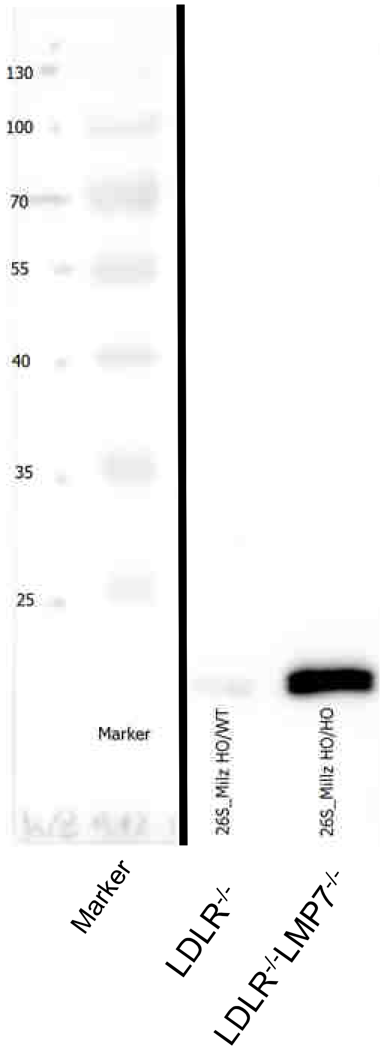

26S BMDM: β1

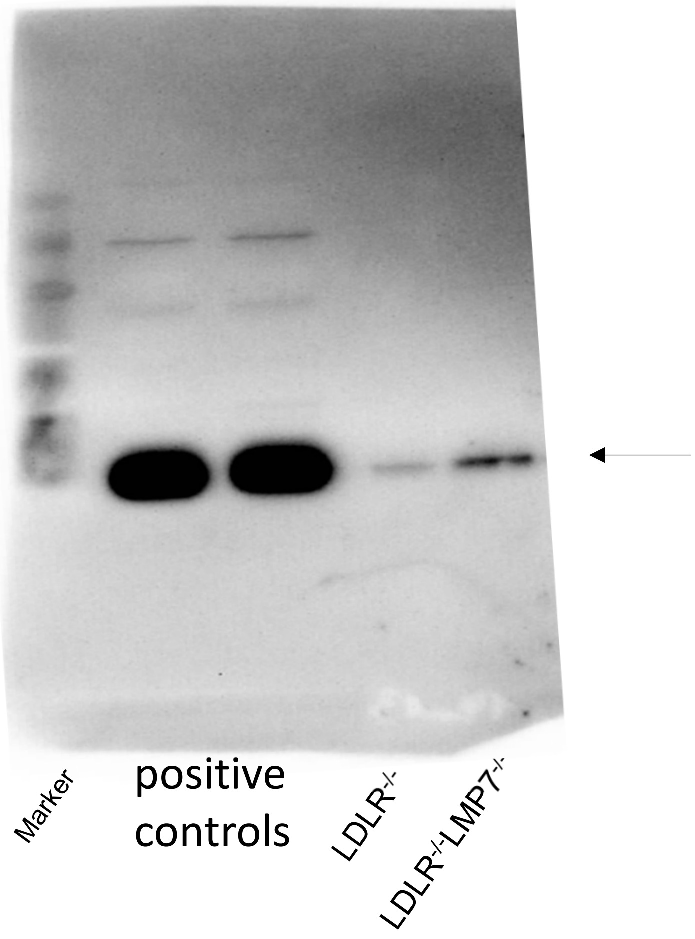

26S spleen: β1

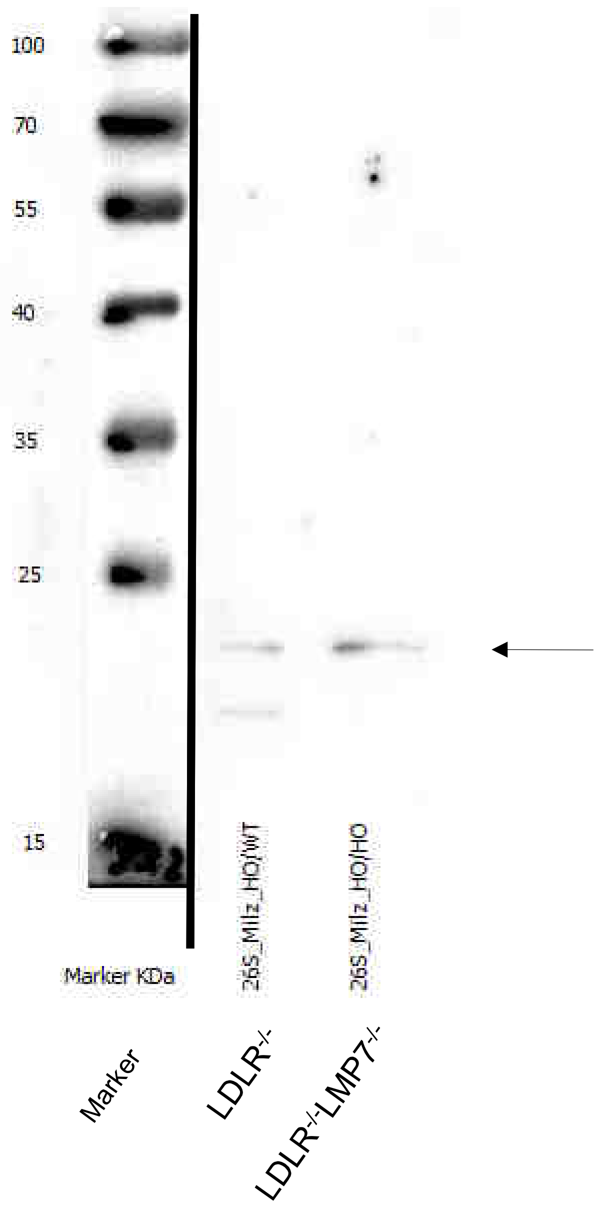

26S BMDM: β2

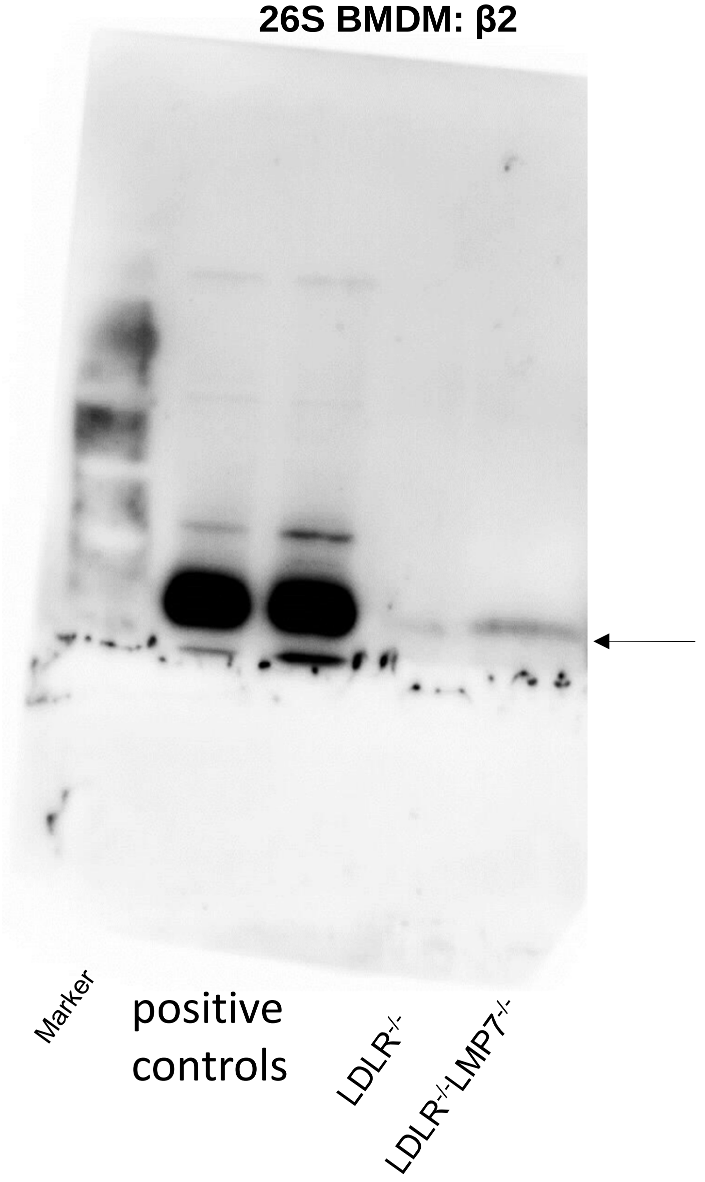

26S spleen: β2

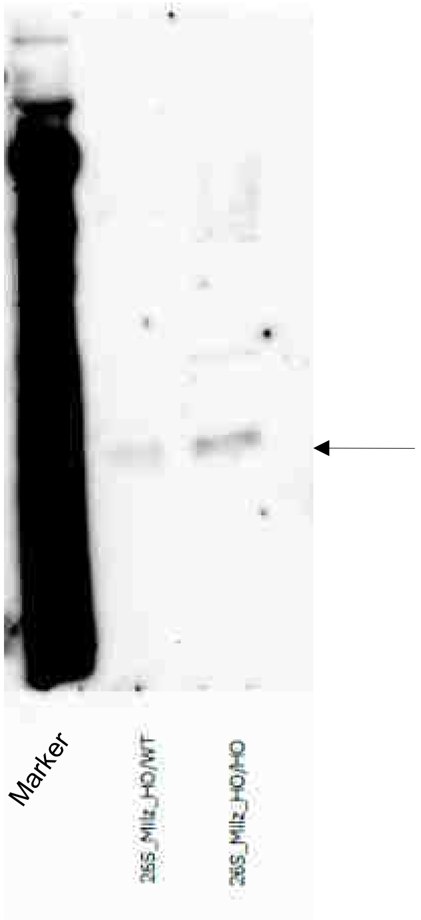

26S BMDM: β5i

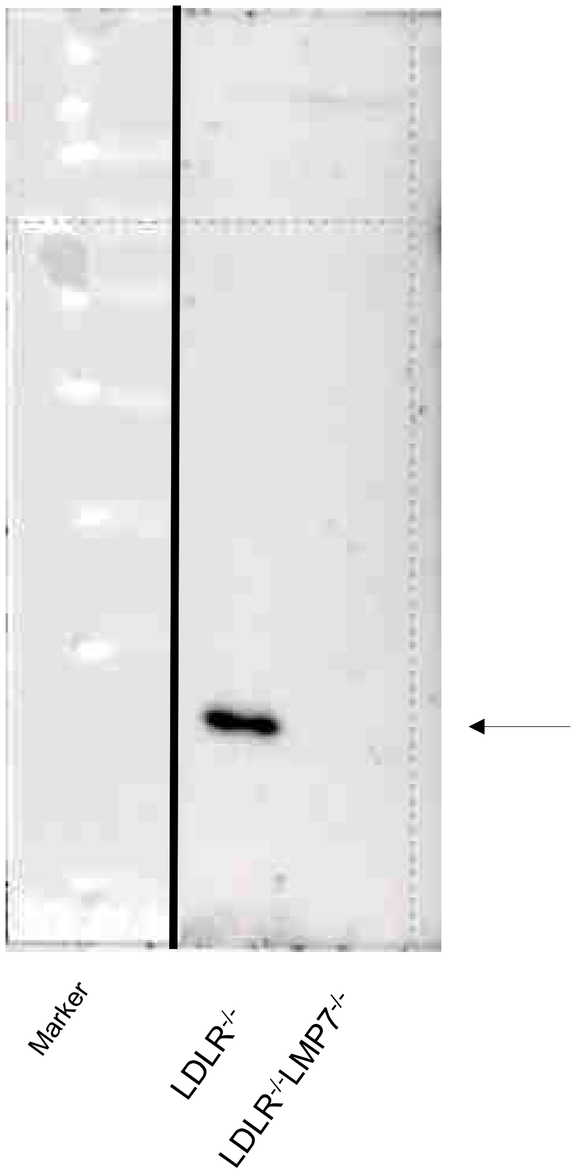

26S spleen: β5i

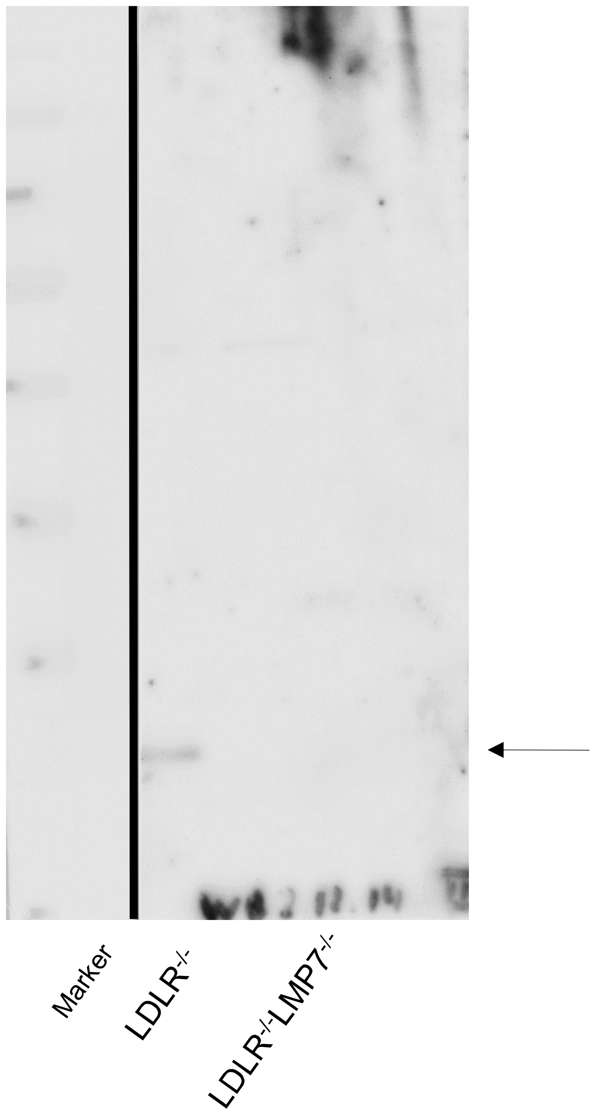

26S BMDM: β1i

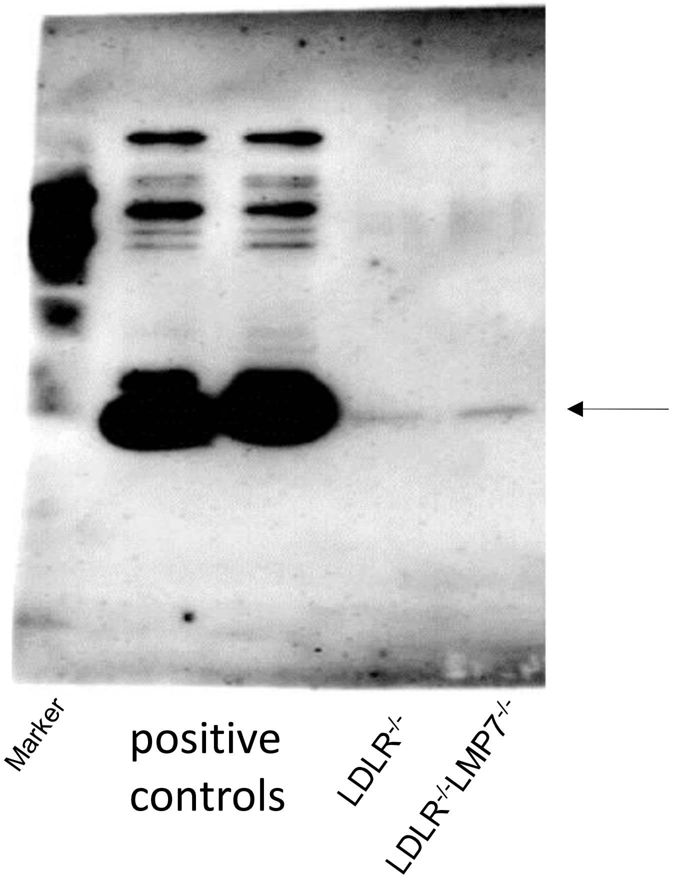

26S spleen: β1i

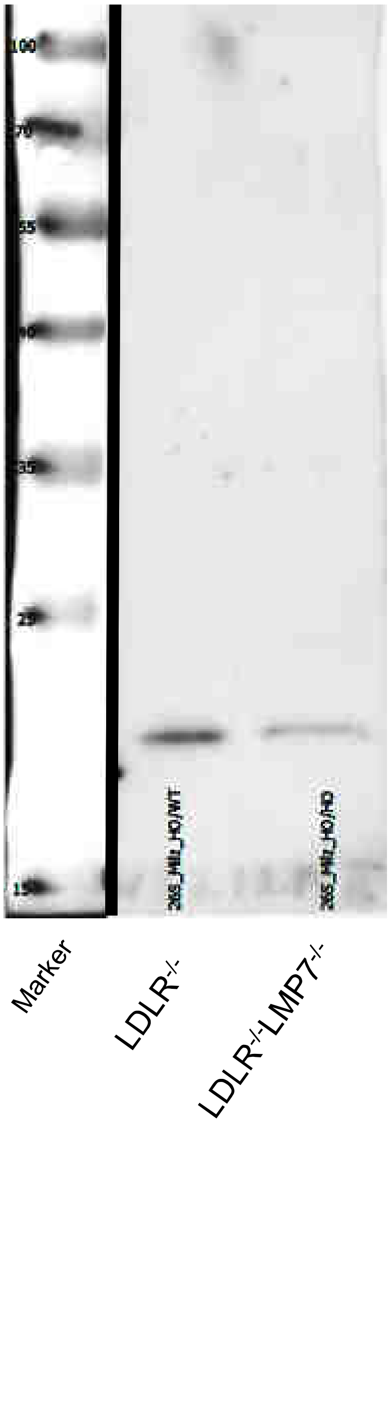

26S BMDM: β2i (~26 kDa)

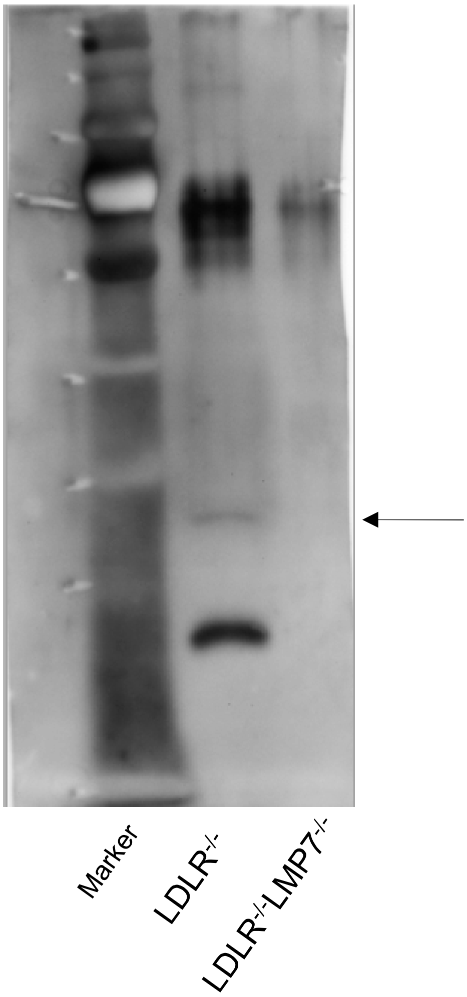

26S spleen: β2i (~26 kDa)

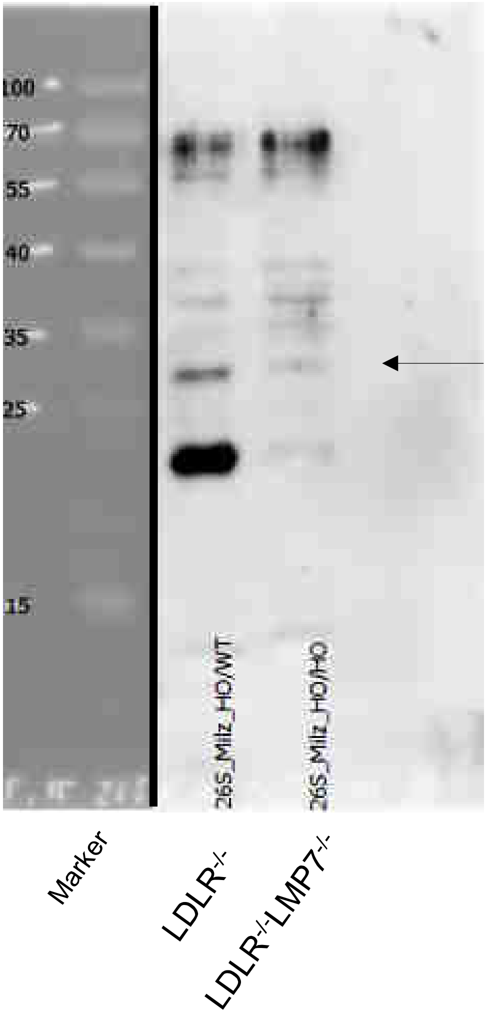

Supplement: Supplementary file 2 — Full-length Western Blots [file 41598_2017_13592_MOESM2_ESM.pdf]
